# Does Evidence Support the American Heart Association's Recommendation to Screen Patients for Depression in Cardiovascular Care? An Updated Systematic Review

**DOI:** 10.1371/journal.pone.0052654

**Published:** 2013-01-07

**Authors:** Brett D. Thombs, Michelle Roseman, James C. Coyne, Peter de Jonge, Vanessa C. Delisle, Erin Arthurs, Brooke Levis, Roy C. Ziegelstein

**Affiliations:** 1 Department of Psychiatry, McGill University, Montréal, Quebéc, Canada; 2 Department of Epidemiology, Biostatistics, and Occupational Health, McGill University, Montréal, Quebéc, Canada; 3 Department of Medicine, McGill University, Montréal, Quebéc, Canada; 4 Department of Educational and Counselling Psychology, McGill University, Montréal, Quebéc, Canada; 5 School of Nursing, McGill University, Montréal, Quebéc, Canada; 6 Lady Davis Institute for Medical Research, Jewish General Hospital, Montréal, Québec, Canada; 7 Behavioral Oncology Program, Abramson Cancer Center and Department of Psychiatry, University of Pennsylvania School of Medicine, Philadelphia, Pennyslvania, United States of America; 8 Health Psychology Section, Department of Health Sciences, University Medical Center Groningen, University of Groningen, Groningen, The Netherlands; 9 Interdisciplinary Center for Psychiatric Epidemiology, University Medical Center Groningen, University of Groningen, Groningen, The Netherlands; 10 Department of Medicine, Johns Hopkins University School of Medicine, Baltimore, Maryland, United States of America; Federal University of Rio de Janeiro, Brazil

## Abstract

**Objectives:**

To systematically review evidence on depression screening in coronary heart disease (CHD) by assessing the (1) accuracy of screening tools; (2) effectiveness of treatment; and (3) effect of screening on depression outcomes.

**Background:**

A 2008 American Heart Association (AHA) Science Advisory recommended routine depression screening in CHD.

**Methods:**

CINAHL, Cochrane, EMBASE, ISI, MEDLINE, PsycINFO and SCOPUS databases searched through December 2, 2011; manual journal searches; reference lists; citation tracking; trial registries. Included articles (1) compared a depression screening instrument to a depression diagnosis; (2) compared depression treatment to placebo or usual care in a randomized controlled trial (RCT); or (3) assessed the effect of screening on depression outcomes in a RCT.

**Results:**

There were few examples of screening tools with good sensitivity and specificity using a priori-defined cutoffs in more than one patient sample among 15 screening accuracy studies. Depression treatment with antidepressants or psychotherapy generated modest symptom reductions among post-myocardial infarction (post-MI) and stable CHD patients (N = 6; effect size = 0.20–0.38), but antidepressants did not improve symptoms more than placebo in 2 heart failure (HF) trials. Depression treatment did not improve cardiac outcomes. No RCTs investigated the effects of screening on depression outcomes.

**Conclusions:**

There is evidence that treatment of depression results in modest improvement in depressive symptoms in post-MI and stable CHD patients, although not in HF patients. There is still no evidence that routine screening for depression improves depression or cardiac outcomes. The AHA Science Advisory on depression screening should be revised to reflect this lack of evidence.

## Introduction

Major depressive disorder (MDD) is present in approximately 20% of coronary heart disease (CHD) patients [1] and is associated with poorer cardiac prognosis [2]. A 2008 American Heart Association (AHA) Science Advisory recommended routine depression screening of all CHD patients [3]. Screening is reasonably considered for important and prevalent conditions that can be effectively treated, but are not readily detected without screening. For screening to be recommended, benefits in excess of potential harms should be demonstrated in well-conducted randomized controlled trials (RCTs) [4]. The AHA recommendation, however, was not based on a systematic review of evidence of likely benefits and harms of the recommended screening intervention, and a systematic review published one month after the Science Advisory reported that no trials had tested whether depression screening in CHD improved patient outcomes [5].

The AHA invests considerable resources in ensuring that practice guidelines are revised rapidly to reflect new evidence [6]. Providing current evidence-based guidelines also requires that recommendations not based on sufficient evidence are revised, and the AHA has done this on a number of occasions [7]. The objective of the present systematic review was to determine whether evidence has been accrued in the last 4 years that would support the AHA Science Advisory on depression screening or whether the Science Advisory should be revised. Review questions included:

**Key Question #1:** What is the accuracy of depression screening instruments in CHD?

**Key Question #2:** Does treatment of depression in CHD improve depressive symptoms or cardiac outcomes?

**Key Question #3:** Does depression screening in CHD improve depression outcomes?

## Methods

This systematic review updates a previous review from November 2008 [5]. Detailed methods were registered in the PROSPERO prospective register of systematic reviews (CRD42011001670).

### Search strategy

To update the previous review [5], we searched the CINAHL, Cochrane, EMBASE, ISI, MEDLINE, PsycINFO and SCOPUS databases from January 1, 2008 through December 2, 2011 (File S1). One search sought studies of screening accuracy (Key Question #1), and a second sought RCTs of depression treatment (Key Question #2) and screening (Key Question #3). Additional searching included reference lists and forward citation of included articles, relevant systematic reviews (File S2), selected journals (December 2011–April 2012; File S3), and trial registries.

### Identification of eligible studies

Eligible articles were original studies in any language with data on adult patients in cardiovascular care settings based on diagnosis or procedure, including mixed populations if CHD data were reported separately. Eligible diagnostic accuracy studies (Key Question #1) reported data allowing determination of sensitivity, specificity, positive predictive value, and negative predictive value compared to a *Diagnostic and Statistical Manual of Mental Disorders* diagnosis of MDD or an *International Classification of Diseases* depressive episode, established with a validated diagnostic interview administered within 2 weeks of the screening tool. Eligible articles for Key Question #2 were RCTs comparing depression treatment with placebo or usual care among CHD patients with MDD or an *International Classification of Diseases* depressive episode based on a validated diagnostic interview. For trials of patients with MDD and other conditions (e.g., minor depression), we sought original study data for patients with MDD for trials with 80% power to detect a 0.50 standardized mean difference effect size (n = 64 per group). Eligible articles for Key Question #3 were RCTs that compared depression outcomes between CHD patients who underwent depression screening and those who did not.

Two investigators independently reviewed titles/abstracts for eligibility with full-text review of articles identified as potentially eligible by one or both. Disagreements after full-text review were resolved by consensus. Chance-corrected agreement was assessed with Cohen's κ.

### Evaluation of eligible studies

Two investigators independently extracted study data (File S4) and assessed risk of bias with discrepancies resolved by consensus. Risk of bias was assessed with the revised Quality Assessment for Diagnostic Accuracy Studies tool for Key Question #1 (File S5) and the Cochrane Risk of Bias tool for Key Question #2 (File S6).

### Data presentation and synthesis

For Key Question #1 (diagnostic accuracy), data were extracted based on optimal cutoffs identified by study authors. For Key Question #2 (treatment) when multiple outcomes were reported, designated primary outcomes were prioritized, followed by observer-rated scales, then self-report measures. Post-intervention effect sizes were reported using the Hedges's *g* statistic (standardized difference between 2 means). The most comprehensive cardiovascular outcome available was extracted.

For Key Question #1, studies were heterogeneous in terms of patient samples, screening tools and cutoffs, and whether they used standard scoring thresholds versus sample-specific thresholds based on exploratory data analysis. For Key Question #2, studies had heterogeneous patient samples, therapeutic interventions, and treatment durations. No eligible studies were identified for Key Question #3. Thus, results were not pooled quantitatively.

## Results

### Key Question #1: Diagnostic Accuracy

**Figure 1:**
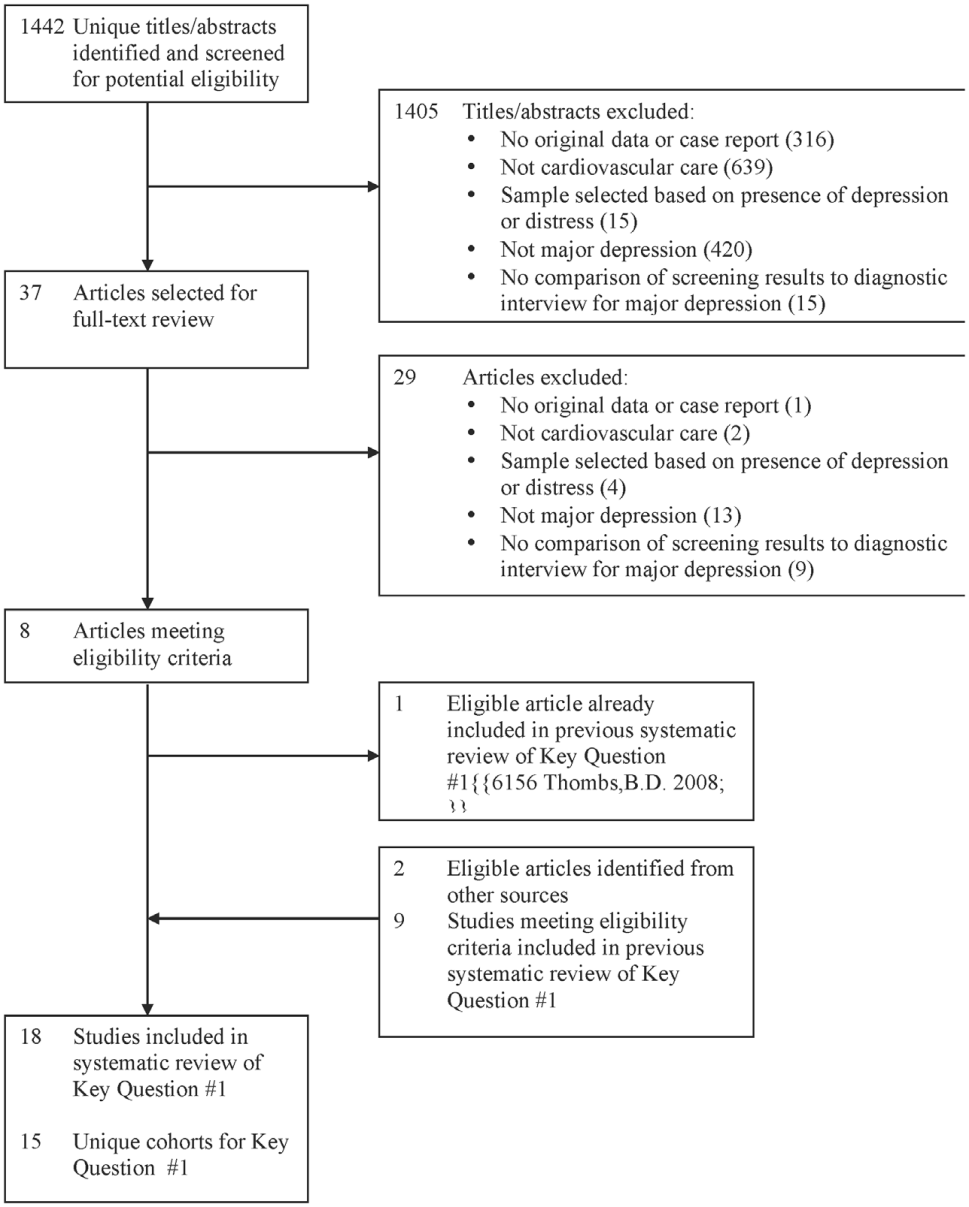
PRISMA Flow Diagram of Study Selection Process for Key Question #1.

Of 1,442 citations, 1,405 were excluded after title/abstract review and 29 after full-text review, leaving 8 eligible articles (Figure 1; κ = 1) [8]–[15], although one [9] was included in the previous review. Two additional articles were identified through other methods [16], [17]. Adding these to 9 from the previous review [9], [18]–[25] resulted in 18 articles [8]–[25] on 15 unique cohorts (Table 1). Two studies [26], [27] from the previous review were excluded because they did not meet the revised eligibility criterion of ≤2 weeks between screening tool and diagnostic interview administration for all patients.

**Table 1:** Characteristics of Studies of Diagnostic Accuracy.

First Author, Year, Country	Setting/Diagnosis	N	Mean Age (Years)	Males (%)	Major Depression Criterion Standard	N (%) Major Depression	Instrument/Cutoff	Derivation of Cutoff	Range of Cutoffs Reported	Sensitivity (%, 95% CI)	Specificity (%, 95% CI)	Positive Predictive Value (%, 95% CI)	Negative Predictive Value (%, 95% CI)
Bunevicius, 2012, Lithuania [17]	Post-ACS Inpatient Rehabilitation	522	58	72	MINI	56 (11%)	HADS-D≥5	Exploratory	≥4–8	77 (64 to 86)	69 (65 to 73)	23 (17 to 29)	96 (93 to 98)
							HADS-A≥8	Exploratory	≥7–9	86 (74 to 93)	72 (67 to 76)	27 (21 to 34)	98 (95 to 99)
							HADS≥14	Exploratory	≥13–15	82 (70 to 90)	79 (75 to 82)	32 (25 to 40)	97 (95 to 99)
							BDI-II≥14	Exploratory	≥13–15	89 (79 to 95)	74 (70 to 78)	29 (23 to 36)	98 (96 to 99)
Cruz, 2010, Brazil [8]	Outpatient IHD	103	61	58	MINI	15 (15%)	BDI≥12	Literature	≥12	100 (80 to 100)	72 (61 to 80)	38 (24 to 53)	100 (94 to 100)
Dickens, 2004, United Kingdom [18]	Inpatient Post-AMI	314	58	63	SCAN	65 (21%)	HADS≥17	Exploratory	≥17	88 (78 to 94)	85 (80 to 89)	60 (50 to 69)	96 (93 to 98)
Frasure-Smith, 1995, Canada [19] *	Inpatient Post-AMI	218	60	78	Modified DIS†	33 (15%)	BDI≥10	Literature	≥10	82 (66 to 91)	78 (71 to 83)	40 (29 to 52)	96 (92 to 98)
Frasure-Smith, 2008, Canada [9]	Outpatient Post-ACS	804	60	81	SCID-IV	57 (7%)	BDI-II≥14	Literature	≥14	91 (81 to 96)	78 (74 to 80)	24 (19 to 30)	99 (98 to 100)
							HADS-A≥8	Literature	≥8	84 (73 to 91)	62 (58 to 65)	14 (11 to 19)	98 (96 to 99)
Freedland, 2003, United States [20]	Inpatient CHF	613	66‡	49	Modified DIS§	120 (20%)	BDI≥10	Literature	≥10	88 (80 to 92)	58 (54 to 63)	34 (29 to 39)	95 (92 to 97)
Gutierrez, 1999, Canada [21]	Outpatient CHF	40	70	50	SCID-IV	6 (15%)	BDI≥13	Unclear	≥13	83 (44 to 97)	94 (81 to 98)	71 (36 to 92)	97 (85 to 99)
**Heart and Soul**													
McManus, 2005, United States [22]	Outpatient CHD	1,024	67	82	DIS	224 (22%)	CES-D-10≥10	Literature	≥10	76 (70 to 81)	79 (76 to 82)	50 (45 to 56)	92 (90 to 94)
							PHQ-9≥10	Literature	≥10	54 (47 to 60)	90 (88 to 92)	60 (53 to 67)	87 (85 to 90)
							PHQ-2≥3	Literature	≥3	39 (33 to 45)	92 (90 to 94)	58 (50 to 65)	84 (82 to 87)
							2-item yes/no PHQ≥1	Literature	≥1	90 (86 to 93)	69 (66 to 72)	45 (40 to 50)	96 (94 to 97)
Thombs, 2008, United States [11]		1,024					PHQ-9≥6	Exploratory	≥4–10	83 (78 to 87)	76 (73 to 79)	50 (45 to 55)	94 (92 to 96)
							PHQ-2≥2	Exploratory	≥1–3	82 (76 to 86)	79 (76 to 81)	52 (46 to 57)	94 (92 to 95)
							PHQ-2≥2 followed by PHQ-9≥6	Exploratory	PHQ-2≥2 followed by				
PHQ-9≥6	75 (69 to 81)	84 (81 to 86)	57 (51 to 62)	92 (90 to 94)									
							“PHQ diagnosis”∥	Literature	“PHQ diagnosis”∥	28 (22 to 34)	96 (94 to 97)	65 (55 to 73)	83 (80 to 85)
Elderon, 2011, United States [10]		1,022					AHA protocol: 2-item yes/no PHQ≥1, followed by PHQ-9≥10	Literature	2-item yes/no PHQ≥1, followed by PHQ-9≥10¶	52 (46 to 59)	91 (89–93)	63 (56 to 70)	87 (85 to 89)
							PHQ-2≥2 followed by PHQ-9≥10	Exploratory	PHQ-2≥2 followed by PHQ-9≥10¶	50 (44 to 57)	92 (90 to 94)	63 (56 to 70)	87 (84 to 89)
**Huffman**													
Huffman, 2006, United States [23]	Inpatient												
Post-AMI	131	62	80	SCID-IV	17 (13%)	2-items from BDI (item 1+ item 12)≥1	Exploratory	5 different item combinations	94 (73 to 99)	76 (68 to 83)	37 (24 to 52)	99 (94 to 100)	
Huffman, 2010, United States [12]							BDI-II≥16	Exploratory	≥10–19	88 (66 to 97)	92 (86 to 96)	63 (43 to 79)	98 (93 to 99)
							BDI-II cognitive subscale≥3#	Exploratory	≥1–5	88 (66 to 97)	82 (73 to 88)	42 (27 to 58)	98 (93 to 99)
Jacq, 2009, France [13]	Outpatient ICD recipients	65	60	86	MINI	14 (22%)	HADS-D≥8	Literature	≥8	57 (33 to 79)	90 (79 to 96)	62 (36 to 82)	88 (77 to 95)
Low, 2007, Canada [24]	Outpatient Post-ACS	112	63	75	SCID-IV	7 (6%)	BDI-II≥10**	Exploratory	≥9–17	100 (65 to 100)	75 (66 to 83)	21 (11 to 38)	100 (95 to 100)
		119					GDS≥13**	Exploratory	≥9–14	100 (65 to 100)	91 (84 to 95)	41 (22 to 64)	100 (96 to 100)
Pinho,†† 2010, Brazil [14]	Outpatient CHD	209	77	52	CAMDEX	38 (27%)	GDS-15≥7	Exploratory	≥1–15	87 (73 to 94)	75 (66 to 83)	58 (45 to 70)	94 (86 to 97)
Stafford, 2007, Australia [25]	Outpatient Post-AMI, CABG, or PTCA	193	64	81	MINI	35 (18%)	HADS-D≥6	Exploratory	≥5, ≥6, ≥8	80 (64 to 90)	82 (75 to 87)	49 (37 to 62)	95 (90 to 97)
							PHQ-9≥6	Exploratory	≥5, ≥6, ≥10, DSM-IV algorithm	83 (67 to 92)	78 (71 to 84)	46 (34 to 58)	95 (90 to 98)
Swardfager,†† 2011, Canada [16]	Outpatient CHD in cardiac rehabilitation	195	64	80	SCID-IV	43 (22%)	CES-D≥16	Literature	≥16	93 (81 to 98)	93 (88 to 96)	78 (65 to 88)	98 (94 to 99)
Tiringer,†† 2008, Hungary [15]	Post-AMI or post-procedure in inpatient rehabilitation	218	62‡‡	67‡‡	MINI-Plus	19 (9%)	HADS-D≥9	Exploratory	≥9	91 (72 to 97)	83 (77 to 87)	37 (25 to 50)	99 (96 to 100)

* Corrected diagnostic accuracy data were provided in a subsequent erratum (Frasure-Smith N, Lesperance F, Talajic M. Depression after myocardial infarction: Response. *Circulation*. 1998; 97: 707–708).

† The modified DIS did not require that symptoms be of at least 2 weeks duration and did not apply the criteria of seeking medical help and experiencing impairment.

‡ Mean age based on full study sample of 682 patients, rather than the 613 patients included in the analyses reported in the table.

§ The depression section of the modified DIS starts with somatic rather than cognitive or mood-related symptoms and focuses on current rather than lifetime symptoms.

∥ Patients met the “PHQ diagnosis” if they reported a total of 5 of 9 PHQ symptoms, including anhedonia or depressed mood, more than half the days in the past 2 weeks.

¶ Diagnostic data were also reported for the 2-item yes/no PHQ≥1 alone and the PHQ-9≥10 alone, which were already reported for this cohort in McManus, 2005 [22], and for the PHQ-2≥2, which was already reported for this cohort in Thombs, 2008 [11].

# The cutoffs of ≥3 and ≥4 on the BDI-II cognitive subscale were both identified as optimal in the published manuscript. The diagnostic data for the BDI-II cognitive subscale ≥4 are sensitivity 82% (95% CI: 59% to 94%), specificity 89% (95% CI: 81% to 93%), positive predictive value 52% (95% CI: 34% to 69%), and negative predictive value 97% (95% CI: 92% to 99%).

** Diagnostic data were reported for the literature-based cutoffs of BDI≥14 and GDS≥11 in the previous systematic review [5]. However, the cutoffs of BDI≥10 and GDS≥13 were identified as optimal by the study authors.

†† The diagnostic data were provided by the authors of the original studies to correct inconsistencies in the published manuscripts.

‡‡ Demographic data based on full study sample of 747 patients, rather than the 218 patients included in the analyses reported in the table. ACS = acute coronary syndrome; AHA = American Heart Association; AMI = acute myocardial infarction; BDI = Beck Depression Inventory; BDI-II = Beck Depression Inventory - II; CABG = coronary artery bypass graft surgery; CAMDEX = Cambridge Examination for Mental Disorders of the Elderly; CES-D = Center for Epidemiological Studies Depression Scale; CES-D-10 = 10-item version of the Center for Epidemiological Studies Depression Scale; CHD = coronary heart disease; CHF = congestive heart failure; CI = confidence interval; DIS = Diagnostic Interview Schedule; GDS = Geriatric Depression Scale; HADS = Hospital Anxiety and Depression Scale, total score; HADS-A = Anxiety Subscale of the Hospital Anxiety and Depression Scale HADS-D = Depression Subscale of the Hospital Anxiety and Depression Scale; ICD = implantable cardioverter defibrillator; IHD = ischemic heart disease; MINI = Mini International Neuropsychiatric Interview; MINI-Plus = extended version of Mini International Neuropsychiatric Interview; PHQ-2 = Patient Health Questionnaire - 2; PHQ-9 = Patient Health Questionnaire - 9; PTCA = percutaneous transluminal coronary angioplasty; SCAN = Schedule for Assessment of Neuropsychiatric Disorders; SCID-IV = Structured Clinical Interview for DSM-IV.

Sample sizes in the 15 cohorts ranged from 40 to 1,024 (median = 209) and MDD cases from 6 to 224 (median = 35). Diagnostic accuracy was based on a standard cutoff score for 6 cohorts [8], [9], [13], [16], [19], [20], on exploratory methods for 7 [12], [14], [15], [17], [18], [23]–[25], not specified in 1 [21], and on both exploratory methods and standard cutoffs in different articles for 1 cohort [10], [11], [22].

Two studies tested the standard cutoff of ≥10 on the Beck Depression Inventory. One reported good sensitivity (82%) and specificity (78%) post-myocardial infarction (post-MI) [19], whereas the other reported good sensitivity (88%) but poor specificity (58%) with hospitalized heart failure (HF) patients [20]. For the Beck Depression Inventory-II, two studies [9], [17] reported good sensitivity (89–91%), but lower specificity (74–78%) based on the standard cutoff of ≥14. In one cohort [11], [22], the Patient Health Questionnaire (PHQ-9) had poor sensitivity (54%), but good specificity (90%) with a standard cutoff of ≥10, and a cutoff score of ≥6 maximized sensitivity (83%) and specificity (76%), consistent with results from another cohort [25].

Risk of bias was unclear or high for 10 of 18 articles that did not pre-specify a cutoff for the screening test. With one exception [17], no studies excluded already diagnosed or treated patients who would not be screened to identify new cases in clinical settings (File S7).

### Key Question #2: Treatment

**Figure 2:**
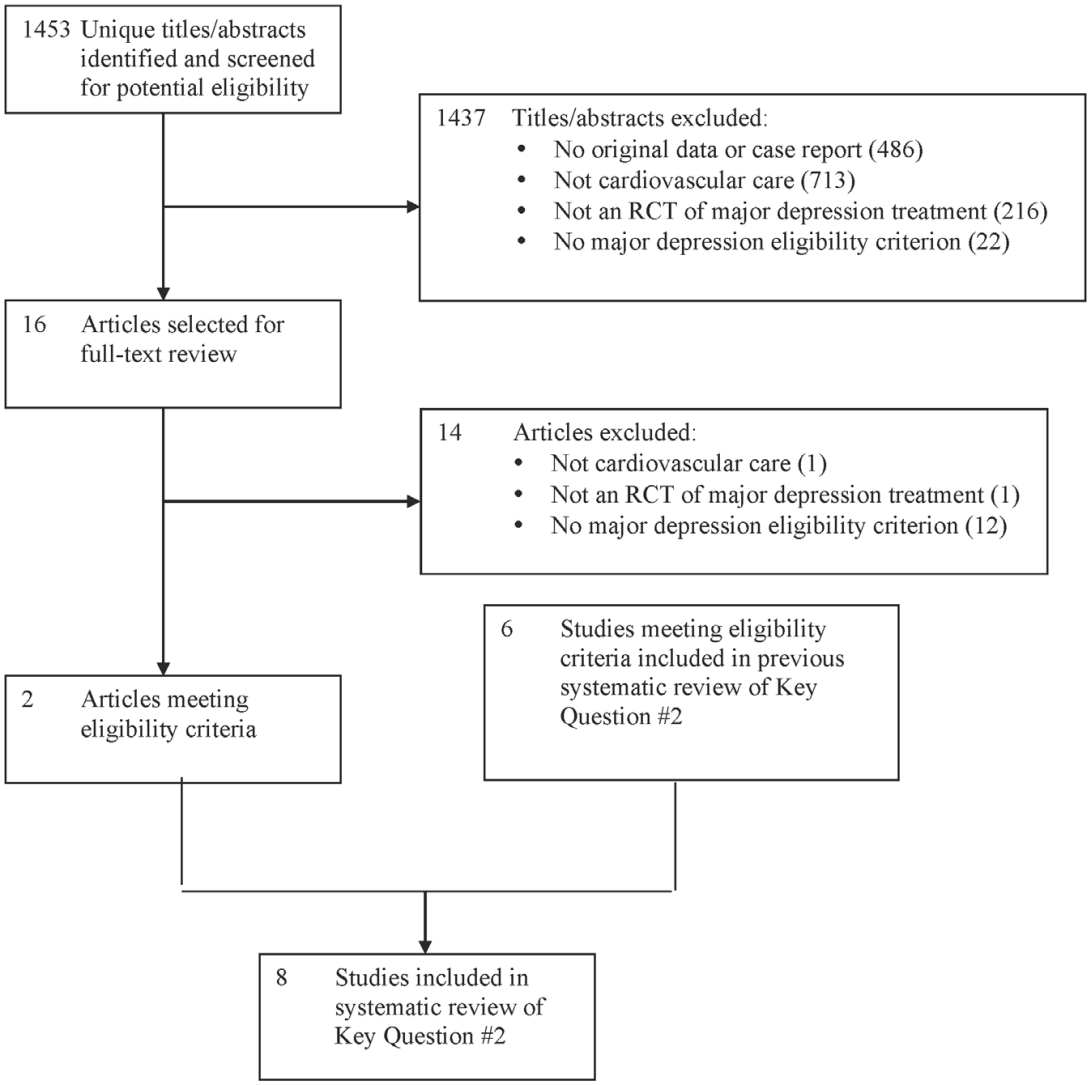
PRISMA Flow Diagram of Study Selection Process for Key Question #2.

Of 1,453 unique titles/abstracts, 1,437 were excluded after title/abstract review and 14 after full-text review, leaving 2 eligible RCTs (Figure 2; κ = 1) [28], [29]. With 6 studies from the previous review [30]–[35], there were 8 treatment trials (Table 2).

**Table 2:** Characteristics of Randomized Controlled Trials of Depression Treatment.

Trial, Year, Site(s)	Diagnosis	Timing of Assessment Relative to Acute Event	Treatment vs. Control	N Randomized	Mean Age (Years)	Males (%)	Treatment Duration	Follow-up Duration for Cardiovascular Events from Randomization	Trial Registration Requirement,* Registration Status, Registration Number	Study Funding Source	Number of Authors with Disclosed Conflicts of Interest/Total Number of Authors
CREATE, 2007† Canada [30]	CHD	NA	Citalopram vs. Placebo	Total = 284; Tx = 142; Placebo = 142	Tx = 58; Placebo = 58	Tx = 77%; Placebo = 74%	12 weeks	12 weeks	Registration required, registered during ongoing recruitment, ISRCTN15858091	Non-industry, study drug supplied by industry	6/11
	CHD	NA	IPT+ CM vs. CM	Total = 284; Tx = 142; CM only = 142	Tx = 59; CM only = 57	Tx = 69%; CM only = 82%	12 weeks	12 weeks			
ENRICHD, 2003,‡ United States [31]	Post-AMI	≤28 days	CBT vs. UC	Total = 955; Tx = 474; UC = 481	Tx = 59; UC = 59	Tx = 50%; UC = 51%	26 weeks§	18–48 months	Pre-requirement, registered post-hoc, NCT00000557	Non-industry	NR
Fraguas, 2009, Brazil [28]	HF	NA	Citalopram vs. Placebo	Total = 37; Tx = 19; Placebo = 18	Tx = 74; Placebo = 73	Tx = 53%; Placebo = 44%	8 weeks	NA	Pre-requirement, not registered, NA	Non-industry	NR
Honig, 2007,∥ Netherlands [32]	Post-AMI	3–12 months	Mirtazapine vs. Placebo	Total = 91; Tx = 47; Placebo = 44	Tx = 57; Placebo = 58	Tx = 87%; Placebo = 82%	8 weeks¶	24 weeks	Pre-requirement, not registered, NA	Combined industry and non-industry	NR#
MIND-IT, 2007,∥ Netherlands [33]	Post-AMI	3–12 months	Active Treatment vs. UC	Total = 331; Tx = 209; UC = 122	Tx = 59; UC = 58	Tx = 76%; UC = 74%	24 weeks	6–15 months	Pre-requirement, not registered, NA	Combined industry and non-industry	0/10
SADHART, 2002, United States, Canada, Europe, Australia [34]	Post-ACS	1^st^≤30 days followed by 2^nd^ assessment after 2-week placebo run-in	Sertraline vs. Placebo	Total = 369; Tx = 186; Placebo = 183	Tx = 57; Placebo = 58	Tx = 63%; Placebo = 64%	24 weeks	(1) 24 weeks(2) median 6.7 years**	Pre-requirement, not registered, NA	Combined industry and non-industry	9/15, including two authors employed by industry
SADHART-CHF, 2010, United States [29]	HF	NA	Sertraline vs. Placebo	Total = 469; Tx = 234; Placebo: = 235	Tx = 63; Placebo = 61	Tx = 62%; Placebo = 57%	12 weeks	12 weeks	Registration required Registered during ongoing recruitment NCT00078286	Non-industry	5/11
Strik, 2000, Netherlands [35]	Post-AMI	3–12 months	Fluoxetine vs. Placebo	Total = 54; Tx = 27; Placebo = 27	Tx = 54; Placebo = 59	Tx = 78%; Placebo = 63%	25 weeks	25 weeks	Pre-requirement, not registered, NA	Combined industry and non-industry	NR

* The International Committee of Medical Journal Editors (ICMJE) clinical trial registration policy requires prospective registration (i.e., prior to patient enrollment) of trials that began on or after July 1, 2005, and also requires registration of trials that were ongoing as of July 1, 2005 (i.e., registration after the beginning of patient enrollment).

† Factorial design with patients randomized to citalopram or placebo and IPT or CM.

‡ Of the 2,481 randomized patients in the ENRICHD trial who met eligibility criteria for MDD, minor depression, or dysthymia and/or low social support [31], data are reported only for the subset of 955 randomized patients diagnosed with MDD at trial entry. Original data for the ENRICHD trial were obtained from the National Heart Lung and Blood Institute.

§ Maximum duration of the CBT intervention was 6 months. Group therapy could extend 12 additional weeks and adjunctive sertraline treatment for up to 12 months.

∥ The Honig, 2007 [32] study was an RCT nested within the MIND-IT study [33].

¶ Outcome at 8 weeks were reviewed instead of 24-week results because 8 weeks open treatment with citalopram was offered in the case of refusal or insufficient treatment response [33].

# Author-industry financial ties were not reported. The authors of the MIND-IT trial [33], of which the Honig, 2007 study [32] was a nested RCT, declared no conflicts of interest. However, of the 12 authors of the Honig, 2007 study [32] only 9 were authors of the published MIND-IT report [33].

** Long-term cardiovascular outcomes from Glassman AH, Bigger JT,Jr, Gaffney M. Psychiatric characteristics associated with long-term mortality among 361 patients having an acute coronary syndrome and major depression: Seven-year follow-up of SADHART participants. Arch Gen Psychiatry. 2009 Sep;66:1022–9. ACS = acute coronary syndrome; AMI = acute myocardial infarction; CBT = cognitive behavior therapy; CHD = coronary heart disease; CM = clinical management; CREATE = Canadian Cardiac Randomized Evaluation of Antidepressant and Psychotherapy Efficacy trial; ENRICHD = Enhancing Recovery in Coronary Heart Disease Patients; HF = heart failure; IPT = interpersonal therapy; MIND-IT = Myocardial Infarction and Depression-Intervention trial; NA = not applicable; NR = not reported; SADHART = Sertraline Antidepressant Heart Attack Randomized trial; SADHART-CHF = Sertraline Against Depression and Heart Disease in Chronic Heart Failure; Tx = treatment; UC = usual care.

There were 6 antidepressant studies [28]–[30], [32], [34], [35] including 3 with post-MI patients that tested mirtazapine (in a RCT nested within the MIND-IT study) [32], sertraline [34], and fluoxetine [35]; 2 with HF patients that tested citalopram [28] and sertraline [29]; and 1 with stable CHD patients that tested citalopram [30]. The 4 studies in post-MI and stable CHD patients all reported positive, albeit small, effects (Hedges's *g* = 0.20–0.38). The 2 studies [28], [29] that treated patients with HF, on the other hand, did not find that citalopram [28] or sertraline [29] reduced symptoms of depression compared to placebo. One small study of citalopram in HF [28], however, was prematurely halted after an unplanned interim analysis of a small number of patients (total N = 37), which showed substantive symptom reduction in both the citalopram and placebo groups. In the other study, the SADHART-CHF trial [29], which enrolled 469 patients, patients in both the sertraline and placebo groups both received nurse-facilitated depression management support in addition to sertraline or placebo.

There were 2 studies where psychotherapy was investigated. The Enhancing Recovery in Coronary Heart Disease Patients (ENRICHD) trial [31], found that cognitive behavior therapy reduced depressive symptoms (Hedges's *g* = 0.20, 95% confidence interval [CI], 0.07 to 0.33). This was compared to usual care. Among depressed patients enrolled in the ENRICHD trial, 28% of patients in the cognitive behavior therapy intervention arm and 21% in the usual care arm were prescribed an antidepressant in the 12 months following trial enrolment. The other psychotherapy trial, the Canadian Cardiac Randomized Evaluation of Antidepressant and Psychotherapy Efficacy trial [30], was a parallel-group, 2×2 factorial trial that compared citalopram to placebo and short-term interpersonal psychotherapy plus clinical management to clinical management alone in patients with CHD. Patients in clinical management alone had lower levels of depressive symptoms than patients who received interpersonal therapy, although this was not statistically significant. In the CREATE trial, patients who received citalopram, with or without interpersonal psychotherapy, were compared to patients who did not receive citalopram, also with or without interpersonal psychotherapy. Similarly, patients who received interpersonal psychotherapy plus clinical management, with or without citalopram, were compared to patients who received only clinical management, with or without citalopram. Clinical management involved 20- to 25-minute visits that included information on depression and antidepressants, reassurance, and encouragement to adhere with medication.

**Figure 3:**
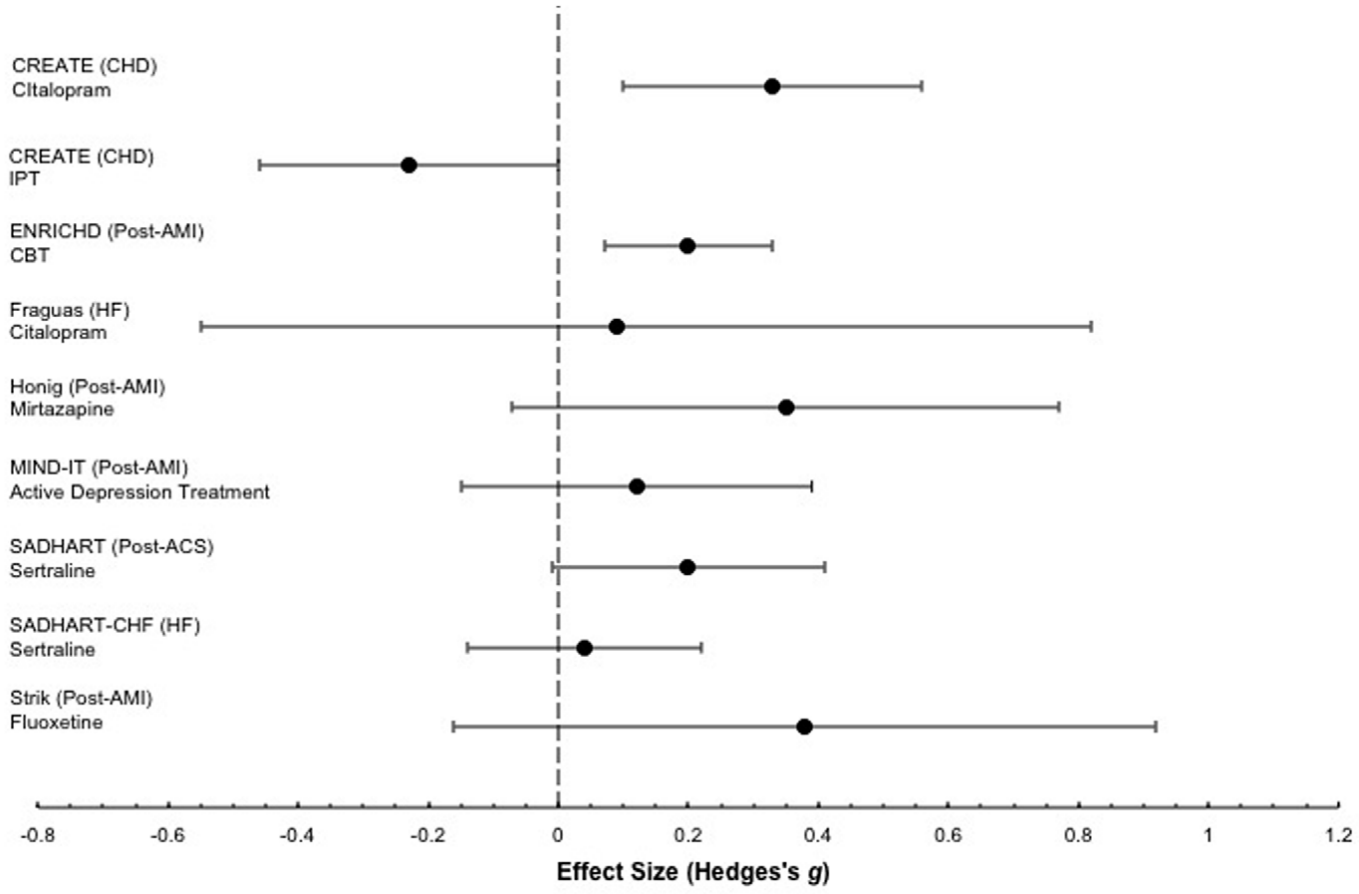
Forest Plot of Effect Sizes of Depression Treatment Studies (Key Question #2).

Figure 3 provides a forest plot of the effect sizes for reductions in depressive symptoms for the primary outcome variables in each treatment study.

No studies reported improved cardiac outcomes, although only ENRICHD [31] and the Myocardial Infarction and Depression–Intervention Trial (MIND-IT) [33] were designed for this purpose, and MIND-IT had very low power (Table 3). MIND-IT was designed as an effectiveness study that compared active depression treatment to usual care. In MIND-IT, among patients not lost to follow-up, 77% of patients randomized to the active depression treatment received depression treatment via enrollment in a nested double-blind mirtazapine versus placebo trial (48%) [32], by receiving open pharmacological treatment (9%), or by receiving non-pharmacological treatment (20%). Patients in the mirtazapine trial who did not respond to mirtazapine treatment or placebo were offered open pharmacological treatment at the end of the 12-week trial period. Thus, there were 18 patients (9%) who were included in the active depression intervention group, but only received placebo as part of the mirtazapine trial. In the usual care group, among patients not lost to follow-up, 17% received pharmacological or non-pharmacological depression treatment outside of the trial.

**Table 3:** Outcomes for Randomized Controlled Trials of Depression Treatment.

Trial, Year, Site(s)	N Randomized	Depression Outcomes*		Cardiovascular Outcomes:Percent with Outcome and Odds Ratio (95% CI)
		Primary Outcome:Hedges's *g* (95% CI)	Secondary Outcome(s):Hedges's *g* (95% CI)	
CREATE, 2007, Canada [30]	Citalopram = 142; Placebo = 142	ΔHAMD-24: 0.33 (0.10 to 0.56)	ΔBDI-II: 0.34 (0.10 to 0.57) ΔHAMD-17: 0.29 (0.05 to 0.52)	Cardiovascular serious adverse events:† Tx = 6 (4%); Placebo = 6 (4%); OR = 1.00 (0.32 to 3.18)
	IPT+ CM = 142; CM only = 142	ΔHAMD-24: −0.23 (−0.46 to 0.00)	ΔBDI-II: 0.10 (−0.13 to 0.34) ΔHAMD-17: −0.22 (−0.46 to 0.01)	Cardiovascular serious adverse events:† Tx = 9 (6%); CM only = 3 (2%); OR = 3.14 (0.83 to 11.83
ENRICHD, 2003,‡ United States [31]	CBT = 474; UC = 481	ΔHAMD-17:§ 0.20 (0.07 to 0.33)	ΔBDI:§ 0.31 (0.19 to 0.44)	Recurrent MI or death from any cause: Tx = 128 (27%); UC = 121 (25%); OR = 1.10 (0.82 to 1.47)
Fraguas, 2009, Brazil [28]	Citalopram = 19; Placebo = 18	ΔHAMD-17: 0.09 (−0.56 to 0.73)	ΔHAMD-31: 0.31 (−0.34 to 0.95)ΔMADRS: 0.58 (−0.08 to 1.24)	NA
Honig, 2007,∥ Netherlands [32]	Mirtazapine = 47; Placebo = 44	ΔHAMD-17: 0.35 (−0.06 to 0.77)	ΔBDI: 0.50 (0.08 to 0.91)ΔSCL-90-D: 0.53 (0.11 to 0.95)ΔCGI-S: 0.83 (0.40 to 1.26)CGI-I: 0.30 (−0.11 to 0.72)	Hospitalization: Tx = 8 (17%); Placebo = 10 (23%); OR = 0.70 (0.25 to 1.97)
MIND-IT, 2007,∥ Netherlands [33]	Active Treatment = 209; UC = 122	BDI: * 0.12 (−0.15 to 0.39)	NA	Total cardiac events:¶ Tx = 27 (14%); UC = 15 (13%); OR = 1.10 (0.56 to 2.16)
SADHART, 2002, United States, Canada, Europe, Australia [34]	Sertraline = 186; Placebo = 183	CGI-I: 0.20 (0.00 to 0.41)	ΔHAMD-17:# 0.14 (−0.06 to 0.35)	(1) Major adverse cardiac events:**; Tx = 32 (17%); Placebo = 41 (22%); OR = 0.72 (0.43 to 1.21) (2) All-cause mortality (n = 361); HR = 0.99 (0.63 to 1.56)††
SADHART-CHF, 2010, United States [29]	Sertraline = 234; Placebo = 235	ΔHAMD-17: 0.04 (−0.14 to 0.22)	NA	All-cause mortality or non-fatal cardiovascular event: Tx = 65 (28%); Control = 70 (30%); OR = 0.91 (0.61 to 1.35)
Strik, 2000, Netherlands [35]	Fluoxetine = 27; Placebo = 27	ΔHAMD-17: 0.38 (−0.16 to 0.92)	NA	Cardiac hospitalization: Tx = 1 (4%); Placebo = 6 (22%); OR = 0.13 (0.02 to 1.21)

* All reported depression outcomes were assessed at the end of the treatment period except MIND-IT [33] where depression outcomes were assessed 18 months post-myocardial infarction (0–9 months after completion of treatment).

† Cardiovascular serious adverse events = myocardial infarction, congestive heart failure, worsening angina, stroke, or other cardiovascular events.

‡ Of the 2,481 randomized patients in the ENRICHD trial who met eligibility criteria for MDD, minor depression, or dysthymia and/or low social support [31], data are reported only for the subset of 955 randomized patients diagnosed with MDD. Original data for the ENRICHD trial were obtained from the National Heart Lung and Blood Institute.

§ In the depression outcome analyses presented, the last-observation-carried-forward approach was applied for missing data. The original published report of the ENRICHD trial [31] reported outcome data for completers. Based on completer data only, Δ HAMD-17: Hedges' *g* = 0.24, 95% CI 0.09 to 0.39 (N = 690, CBT: 348, UC: 342). Δ BDI: Hedges' *g* = 0.36, 95% CI 0.21 to 0.51 (N = 699, CBT: 357, UC: 342).

∥ The Honig, 2007 [32] study was an RCT nested within the MIND-IT study [33].

¶ Total cardiac events include cardiac death, recurrent myocardial infarction, revascularization, heart failure, myocardial ischemia, and ventricular arrhythmia. 17 patients were lost to follow-up (Tx, n = 196; UC, n = 118).

# Patients were assessed with HAMD-17 at 16 weeks, but not 24 weeks.

** Major adverse cardiac events = events involving death or requiring hospitalization.

†† Hazard ratio from Kaplan-Meier analysis, but number of deaths per group not provided for follow-up study (Glassman AH, Bigger JT,Jr, Gaffney M. Psychiatric characteristics associated with long-term mortality among 361 patients having an acute coronary syndrome and major depression: Seven-year follow-up of SADHART participants. Arch Gen Psychiatry. 2009 Sep;66:1022–9). BDI = Beck Depression Inventory; BDI-II = Beck Depression Inventory – II; CBT = cognitive behavior therapy; CM = clinical management; CGI-I = Clinical Global Impression-Improvement; CGI-S = Clinical Global Impression-Severity; CI = confidence interval; CREATE = Canadian Cardiac Randomized Evaluation of Antidepressant and Psychotherapy Efficacy trial; ENRICHD = Enhancing Recovery in Coronary Heart Disease Patients; HAMD-17 = 17-item Hamilton Depression Rating Scale; HAMD-24 = 24-item Hamilton Depression Rating Scale; HAMD-31 = 31-item Hamilton Depression Rating Scale; IPT = interpersonal therapy; MADRS = Montgomery-Asberg Depression Rating Scale; MI = myocardial infarction; MIND-IT = Myocardial Infarction and Depression-Intervention trial; NA = not applicable; SADHART = Sertraline Antidepressant Heart Attack Randomized trial; SADHART-CHF = Sertraline Against Depression and Heart Disease in Chronic Heart Failure; SCL-90-D = depression subscale of the Symptom Checklist 90; Tx = treatment; UC = usual care.

Risk of bias ratings are shown in File S8 for each treatment study. Three trials were rated low risk of bias for most categories [29]–[31], although 2 of the 3 [30]–[31] had higher risk associated with the inability to blind participants and study personnel in trials that involved psychotherapy. MIND-IT [33] was rated low risk in most categories, but only reported depression outcomes 18 months post-MI (0–9 months post-treatment), which may have reduced observed effects of treatment. For the other 4 studies [28], [32], [34], [35], risk of bias was unclear or high across several categories.

### Key Question #3: Depression Screening

**Figure 4:**
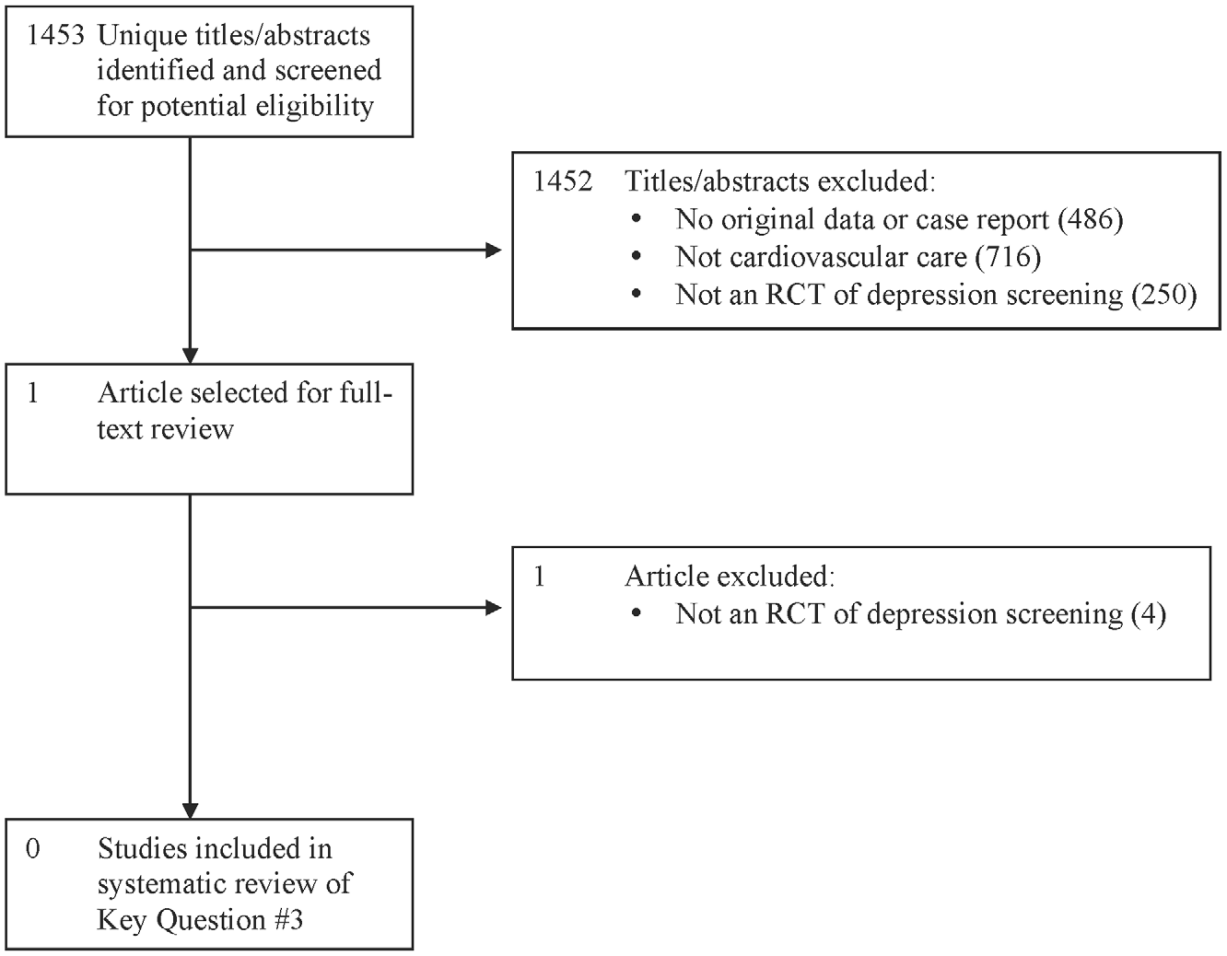
PRISMA Flow Diagram of Study Selection Process for Key Question #3.

Of 1,453 unique titles/abstracts, 1 received full-text review, and no eligible RCTs were identified (Figure 4). Potentially relevant excluded studies are described in File S9.

## Discussion

The main findings of this systematic review are that: (1) there are few examples of screening tools with high sensitivity and specificity using an a priori-defined cutoff score in more than one CHD sample. When results from studies that used a pre-specified score were available in more than one sample, estimates of diagnostic accuracy were inconsistent. When exploratory data analysis methods were used to both generate a cutoff score and assess the accuracy of that cutoff score in the same sample, different studies tended to produce cutoffs that were inconsistent across studies; (2) depression treatment improves symptoms of depression in post-MI and stable CHD patients, although symptom reductions are modest; (3) antidepressant treatment has not reduced depressive symptoms compared to placebo in two trials with HF patients, although one small trial was stopped prematurely, and the other trial provided nurse-facilitated depression management services to patients in both the antidepressant and placebo groups; and (4) no RCTs have evaluated whether routine depression screening in CHD would improve depression outcomes.

The AHA has recommended that all CHD patients be routinely screened for depression [3], but the present systematic review did not find any evidence that depression screening would improve outcomes. This finding is not unique to CHD, since there are no published trials in any patient group where patients screened for depression had better depression outcomes than patients not screened [36]. On the other hand, a 2008 meta-analysis [37] reviewed 11 trials of depression screening in primary care and found several trials where screening increased identification or treatment of depression, but none where screening improved depression outcomes, even though primary care settings are generally much better equipped to manage mental health problems than cardiology settings.

In cardiovascular care settings, several observational studies have reported on the administration of depression questionnaires. One study [38] examined the 2-step protocol recommended by the AHA [3], in which the 2-item PHQ-2 was administered to 3,504 of 4,873 admitted patients, and patients with a positive PHQ-2 screen were administered the PHQ-9. Using this approach, 140 patients were identified as possibly depressed. The study authors concluded that depression screening is feasible, but did not describe the referral and follow-up process, estimate costs, or assess whether benefit was obtained. Other observational studies have reported that implementing screening did not increase recognition of depression compared to settings without screening [39]; that no new, previously unrecognized cases of depression were identified through screening [40]; and that patients with positive depression screens only received follow-up assessments if there was a psychiatrist physically present in the cardiology clinic at the time of the screening, but not if an outpatient psychiatry referral was made [41].

In primary care, the UK's Quality and Outcomes Framework pay-for-performance program introduced routine depression screening for patients with CHD or diabetes in April 2006 [42]. In this context, a retrospective cohort study [42] examined records from April 2007 through March 2008 of 94,570 CHD or diabetes patients from 237 general practices in Scotland, including 1,245 physicians. Of all patients with CHD or diabetes, 67,358 were screened for depression (71%), and 2,269 of those screened (3%) received a new diagnosis of depression or initiated treatment. The number needed to screen was 976 for a new diagnosis of depression and 687 for initiation of antidepressant treatment. Data were not available to determine screening resulted in improved depression outcomes.

The AHA website lists more than 20 patient care guidelines issued since the 2008 AHA Advisory on depression screening [3]. None has recommended that routine screening be implemented as recommended in the Advisory. One guideline statement on secondary prevention [43] cited a 2009 editorial that urged the AHA to reconsider its depression screening recommendation [44] and suggested that depression screening in CHD might be considered, but only if patients have access to case management services in collaboration with their primary care physician and a mental health specialist. This recommendation differs from the AHA Science Advisory, which recommended routine screening followed by referral of all positive screening results for evaluation by a professional qualified in the diagnosis and management of depression. It is closer to the U.S. Preventive Services Task Force depression screening recommendation for primary care [45], which specifies that screening should only occur when integrated depression care systems for evaluation and case management are available. No trials, however, have assessed whether screening in CHD with referral to primary care would benefit patients, and no trials have shown that screening in the context of integrated depression care systems would benefit patients even in primary care [36]. This was an important reason why the UK National Institute of Clinical Excellence [46] did not recommend routine depression screening in primary care. Consistent with this, the authors of the Scottish primary care study [42] concluded that the impact of depression screening, even in terms of case identification and new treatment, were small and that health care systems should carefully consider the resource implications of these programs.

Depression screening can benefit patients only to the extent that it identifies depressed patients not already diagnosed or treated for depression, successfully enrolls those patients in treatment, and achieves positive treatment results. As described recently [36], antidepressant treatment rates are already high and trending upward in CHD patients [47]. By 2002, for instance, 16% of post-MI patients aged 65 and older in Ontario, Canada were prescribed antidepressant medication [48]. Furthermore, existing studies appear to exaggerate the accuracy of depression screening tools due to the inclusion of already diagnosed and treated patients, who would not be screened in clinical practice [49], as well as due to the selective reporting of well-performing results from cutoff scores that generate high levels of accuracy, even though this results in substantially different cutoffs being reported across studies [50]. Finally, treatment of depression is more effective in patients with high levels of symptoms, and most of those who are newly detected via screening would be expected to have less severe symptoms of depression [36].

Without evidence that depression screening benefits CHD patients, the potentially considerable resources and costs that would be involved in implementing routine screening must be even more carefully considered [51]. Practically speaking, costs would include not only administering screening tests to all CHD patients, but also following up on positive depression screens that would be expected in perhaps one-third of all CHD patients [5], even though most would turn out not to be depressed. The optimal interval and associated costs of rescreening must also be considered as ongoing screening would be expected to divert scarce resources away from an overburdened mental health system that already struggles to provide adequate mental health care [36].

Depression screening would almost certainly increase the number of patients using antidepressants, and potential harms of even more widespread use of antidepressants by CHD patients must therefore be considered. Selective serotonin reuptake inhibitors (SSRIs) may act as antiplatelet agents, which can increase the risk of major bleeding, especially when used along with the combination of aspirin and a thienopyridine antiplatelet drug like clopidogrel [52] or in patients taking warfarin [53], [54]. In addition to this risk, many antidepressant drugs inhibit cytochrome P450 isoenzymes and can result in important drug-drug interactions with cardiac medications [55]. For example, an interaction in patients with acute MI between the SSRI paroxetine and the commonly-prescribed beta blocker metoprolol has been described [56]. In addition to the well-recognized cardiac risks of the tricyclic antidepressants [57], the serotonin-norepinephrine reuptake inhibitor antidepressants may increase blood pressure and heart rate [58], and several antidepressant classes may have unfavorable effects on heart rate variability [59]. Reports of potential adverse cardiovascular effects of antidepressant drugs [60]–[64] suggest that additional studies that evaluate cardiovascular side effects of antidepressant drugs in greater numbers of patients followed for longer time periods may be warranted.

The AHA recommendation for depression screening in CHD [3] was made without any evidence that this would improve depression outcomes. The present systematic review shows that, nearly 4 years since the AHA recommendation on depression screening, there is still no evidence that demonstrates that this potentially very costly strategy would benefit patients. There are prior examples where the AHA has recognized the lack of evidence supporting recommendations and, commendably, revised those recommendations [7]. We hope that the AHA will similarly reconsider its recommendation for depression screening of all CHD patients.

## Supporting Information

File S1 **Search Strategies.**
(DOC)

File S2 **Relevant Systematic Reviews.**
(DOCX)

File S3 **Journals Included in Manual Searching.**
(DOCX)

File S4 **Variables Included in Data Extraction Forms.**
(DOCX)

File S5 **QUADAS-2 Risk of Bias and Applicability Judgments.**
(DOCX)

File S6 **Cochrane Risk of Bias Tool Domains.**
(DOCX)

File S7 **Quality Assessment of Studies of Diagnostic Accuracy (QUADAS-2).**
(DOCX)

File S8 **Assessment of Risk of Bias in Randomized Controlled Trials in Key Question #2 (Treatment).**
(DOCX)

File S9 **Excluded Studies for Effect of Screening on Depression Outcomes (Key Question #3).**
(DOCX)
