# Supplementary material for: Does Evidence Support the American Heart Association's Recommendation to Screen Patients for Depression in Cardiovascular Care? An Updated Systematic Review
Source: PLoS One. 2013 Jan 7;8(1):e52654. doi: 10.1371/journal.pone.0052654 (PMC3538724; doi:10.1371/journal.pone.0052654)
Supplement: File S1 — Search Strategies. (DOC) [file pone.0052654.s001.doc]

**SUPPORTING INFORMATION 1. Search Strategies**

**Search Strategies for Key Question #1**

**MEDLINE:**

((“Depressive Disorder”[MeSH] OR “Depression”[MeSH]) AND (“Cardiovascular Diseases”[MeSH] OR “myocardial infarction”[tiab] OR “acute coronary syndrome”[tiab] OR “heart failure”[tiab] OR “CABG”[tiab] OR “coronary artery bypass graft”[tiab] OR “angina”[tiab] OR “coronary artery disease”[tiab] OR “coronary heart disease”[tiab] NOT “stroke”[tiab] NOT “stroke”[MeSH]) AND (“Sensitivity and Specificity”[MeSH] OR “Predictive Value of Tests”[MeSH] OR "statistics and numerical data"[Subheading] OR sensitivity[tiab] OR sensitivities[tiab] OR specificity[tiab] OR specificities[tiab] OR validation[tiab] OR validate[tiab] OR “positive predictive value”[tiab] OR “cut point”[tiab] OR cutoff[tiab] OR cut-off[tiab])

AND (“Psychological Tests”[MeSH] OR “Psychiatric Status Rating Scales”[MeSH] OR “Mass Screening”[MeSH] OR “Interview, Psychological”[MeSH] OR “Self Assessment (Psychology)”[MeSH] OR “Questionnaires”[MeSH] OR screening[tiab] OR screen[tiab] OR questionnaire[tiab] OR questionnaires[tiab] OR inventory[tiab] OR inventories[tiab] OR BDI OR “beck depression inventory” OR bdi-II OR bdi-2 OR “bdi ii” OR “bdi 2” OR HADS OR “hospital anxiety and depression scale” OR CESD OR CES-D OR “Center for epidemiologic studies depression” OR “cardiac depression scale” OR phq OR phq-2 OR “phq 2” OR phq-9 OR “phq 9” OR “patient health questionnaire” OR zds OR “zung depression scale” OR “depression scale”))

**Cochrane:**

#1 MeSH descriptor Sensitivity and Specificity explode all trees

#2 MeSH descriptor Predictive Value of Tests explode all trees

#3 (sensitivity OR sensitivities OR specificity OR specificities OR validation OR validate OR

"positive predictive value" OR "cut point" OR cutoff OR "cut off")

#4 (#1 OR #2 OR #3)

#5 MeSH descriptor cardiovascular diseases explode all trees

#6 (“myocardial infarction” OR “acute coronary syndrome” OR “heart failure” OR “CABG” OR “coronary artery bypass graft” OR “angina” OR “coronary artery disease” OR “coronary heart disease”)

#7 MeSH descriptor Depressive Disorder explode all trees

#8 MeSH descriptor Depression explode all trees

#9 ((#5 OR #6) AND ( #7 OR #8 ))

#10 MeSH descriptor Psychological Tests explode all trees

#11 MeSH descriptor Psychiatric Status Rating Scales explode all trees

#12 MeSH descriptor Mass Screening explode all trees

#13 MeSH descriptor Interview Psychological explode all trees

#14 MeSH descriptor Self Assessment (Psychology) explode all trees

#15 MeSH descriptor Questionnaires explode all trees

#16 (screening OR screen OR questionnaire OR questionnaires OR inventory OR inventories OR BDI OR "beck depression inventory" OR bdi-II OR bdi-2 OR "bdi ii" OR "bdi 2" OR HADS OR "hospital anxiety and depression scale" OR CESD OR CES-D OR "Center for epidemiologic studies depression" OR "cardiac depression scale" OR phq OR phq-2 OR "phq 2" OR phq-9 OR "phq 9" OR "patient health questionnaire" OR zds OR "zung depression scale" OR "depression scale")

#17 (#10 OR #11 OR #12 #13 OR #14 OR #15 OR #16)

#18 (#4 AND #9 AND #17)

**PsycINFO:**

S1 TX (“myocardial infarction” OR “acute coronary syndrome” OR “heart failure” OR “CABG” OR “coronary artery bypass graft” OR “angina” OR “coronary artery disease” OR “coronary heart disease”) or DE "Cardiovascular Diseases"

S2 DE “Major Depression” OR TI depress*

S3 TX (sensitivity OR sensitivities OR specificity OR specificities OR validation OR validate OR "positive predictive value" OR "cut point" OR cutoff OR cut-off)

S4 (DE "Screening Tests" OR DE "Psychological Screening Inventory" OR DE "Inventories" OR DE "Psychiatric Evaluation" OR DE "Psychological Assessment" OR DE "Questionnaires" OR DE "Rating Scales" OR DE "Screening" OR DE "Screening Tests" OR DE "Psychological Screening Inventory" OR TX (screening OR screen OR score OR questionnaire OR questionnaires OR inventory OR inventories OR BDI OR "beck depression inventory" OR bdi-II OR bdi-2 OR "bdi ii" OR "bdi 2" OR HADS OR "hospital anxiety and depression scale" OR CESD OR CES-D OR "Center for epidemiologic studies depression" OR "cardiac depression scale" OR phq OR phq-2 OR "phq 2" OR phq-9 OR "phq 9" OR "patient health questionnaire" OR zds OR "zung depression scale" OR "depression scale"))

S5 S1 AND S2 AND S3 AND S4

**CINAHL:**

S1 ((MH "Depression") OR (MH “Depressive disorders”))

S2 ((MH "Cardiovascular diseases") OR (TX “myocardial infarction” OR “acute coronary syndrome” OR “heart failure” OR “CABG” OR “coronary artery bypass graft” OR “angina” OR “coronary artery disease” OR “coronary heart disease”))

S3 ((MH "Sensitivity and Specificity") OR (MH "False Positive Results") OR (MH "Predictive Value of Tests") OR TX(sensitivity OR sensitivities OR specificity OR specificities OR validation OR validate OR "positive predictive value" OR "cut point" OR cutoff OR cut-off) OR (MH "Reliability and Validity+"))

S4 ((MH "Psychological Tests") OR (MH "Questionnaires+") OR (MH "Psychological Tests") OR (TX (screening OR screen OR score OR questionnaire OR questionnaires OR inventory OR inventories OR BDI OR "beck depression inventory" OR bdi-II OR bdi-2 OR "bdi ii" OR "bdi 2" OR HADS OR "hospital anxiety and depression scale" OR CESD OR CES-D OR "Center for epidemiologic studies depression" OR "cardiac depression scale" OR phq OR phq-2 OR "phq 2" OR phq-9 OR "phq 9" OR "patient health questionnaire" OR zds OR "zung depression scale" OR "depression scale")))

S5 S1 AND S2 AND S3 AND S4

**EMBASE:**

1. (depression OR depressive)
2. (“Cardiovascular disease” OR “myocardial infarction” OR “acute coronary syndrome” OR “heart failure” OR CABG OR “coronary artery bypass graft” OR angina OR “coronary artery disease” OR “coronary heart disease” or “heart” or “cardiac”)
3. (“sensitivity and specificity”/exp OR sensitivity OR sensitivities OR specificity OR specificities OR validation OR validate OR “positive predictive value” OR “cut point” OR cutoff OR cut-off )
4. (screening OR screen OR score OR questionnaire OR questionnaires OR inventory OR inventories OR BDI OR "beck depression inventory" OR bdi-II OR bdi-2 OR "bdi ii" OR "bdi 2" OR HADS OR "hospital anxiety and depression scale" OR CESD OR CES-D OR "Center for epidemiologic studies depression" OR "cardiac depression scale" OR phq OR phq-2 OR "phq 2" OR phq-9 OR "phq 9" OR "patient health questionnaire" OR zds OR zsds OR "zung depression scale" OR “zung self-rating depression scale” OR "depression scale")
5. 1 AND 2 AND 3 AND 4

* Map to preferred terminology, include sub-terms/derivatives (explosion search)

**ISI:**

1. TS=(Depression OR Depressive disorders)
2. TS=(“Cardiovascular disease” OR “cardiovascular diseases” OR “myocardial infarction” OR “acute coronary syndrome” OR “heart failure” OR CABG OR “coronary artery bypass graft” OR “angina” OR “coronary artery disease” OR “coronary heart disease”)
3. TS=(sensitivity OR sensitivities OR specificity OR specificities OR validation OR validate OR “positive predictive value” OR cut point OR cutoff OR cut-off OR “predictive value”)
4. TS=(screening OR screen OR score OR questionnaire OR questionnaires OR inventory OR inventories OR BDI OR “beck depression inventory” OR bdi-II OR bdi-2 OR “bdi ii” OR “bdi 2” OR HADS OR “hospital anxiety and depression scale” OR CESD OR CES-D OR “Center for epidemiologic studies depression” OR “cardiac depression scale” OR phq OR phq-2 OR “phq 2” OR phq-9 OR “phq 9” OR “patient health questionnaire” OR zds OR “zung depression scale” OR “depression scale” OR test OR tests OR interview* OR self-assessment OR self assessment OR evaluat*)
5. #1 AND #2 AND #3 AND #4

* Databases=SCI-EXPANDED, SSCI, A&HCI

**SCOPUS:**

(TITLE-ABS-KEY(depression OR depressive OR depressed)) AND (TITLE-ABS-KEY(“Cardiovascular disease” OR “myocardial infarction” OR “acute coronary syndrome” OR “heart failure” OR CABG OR “coronary artery bypass graft” OR angina OR “coronary artery disease” OR “coronary heart disease”)) AND ((TITLE-ABS-KEY(sensitivity OR sensitivities OR specificity OR specificities OR validation OR validate OR "positive predictive value" OR "cut point" OR cutoff OR cut-off)) OR (TITLE-ABS-KEY("predictive value"))) AND (TITLE-ABS-KEY(screening OR screen OR score OR questionnaire OR questionnaires)) OR (TITLE-ABS-KEY(inventory OR inventories OR bdi OR "beck depression inventory" OR bdi-ii OR bdi-2 OR "bdi ii" OR "bdi 2")) OR (TITLE-ABS-KEY(phq OR phq-2 OR "phq 2" OR phq-9 OR "phq 9" OR "patient health questionnaire" OR zds OR "zung depression scale" OR HADS OR “hospital anxiety and depression scale” OR CESD OR CES-D OR “Center for epidemiologic studies depression” OR “cardiac depression scale”)) OR (TITLE-ABS-KEY("depression scale" OR test OR tests OR interview* OR self-assessment OR “self assessment” OR evaluat*))

**Search Strategies for Key Questions #2 and #3**

**MEDLINE**

("Depressive Disorder"[MeSH] OR "Depression"[MeSH]) AND ("Cardiovascular Diseases"[Mesh] OR “myocardial infarction”[tiab] OR “acute coronary syndrome”[tiab] OR “heart failure”[tiab] OR “CABG”[tiab] OR “coronary artery bypass graft”[tiab] OR “angina”[tiab] OR “coronary artery disease”[tiab] OR “coronary heart disease”[tiab] NOT “stroke”[tiab] NOT “stroke”[MeSH]) AND (Therapeutics [MeSH] OR therapy [tiab] OR “drug therapy” [MeSH] OR intervention [tiab] OR pharmacological [tiab] OR “antidepressive agents” [MeSH] OR antidepress* [tiab] OR SSRI [tiab] OR psychotherapy [MeSH] OR treatment [tiab] OR psychologic [tiab] OR “cognitive therapy” [MeSH] OR “behavior therapy” [MeSH] OR “treatment outcome” [MeSH ] OR “collaborative care” [tiab] OR screen [tiab] OR screening [tiab] OR assessment [tiab] OR evaluation [tiab])

*Limits: Humans, clinical trial, randomized controlled trial

**Cochrane:**

#1 MeSH descriptor Depressive Disorder explode all trees

#2 MeSH descriptor Depression explode all trees

#3 (#1 OR #2)

#4 MeSH descriptor Therapeutics explode all trees

#5 MeSH descriptor Psychotherapy explode all trees

#6 MeSH descriptor Treatment Outcome explode all trees

#7 MeSH descriptor Antidepressive Agents explode all trees

#8 therapy: ti,ab,kw

#9 intervention: ti,ab,kw

#10 pharmacological: ti,ab,kw

#11 antidepress*: ti,ab,kw

#12 SSRI: ti,ab,kw

#13 treatment: ti,ab,kw

#14 psychotherapy: ti,ab,kw

#15 psychological: ti,ab,kw

#16 “collaborative care”: ti, ab, kw

#17: screen: ti, ab, kw

#18: screening: ti, ab, kw

#19: assessment: ti, ab, kw

#20: evaluation: ti, ab, kw

#21 (#4 OR #5 OR #6 OR #7 OR #8 OR #9 OR #10 OR #11 OR #12 OR #13 OR #14 OR

#15 OR #16 OR #17 OR #18 OR #19 OR #20)

#22 MeSH descriptor cardiovascular diseases explode all trees

#23 (“myocardial infarction” OR “acute coronary syndrome” OR “heart failure” OR “CABG” OR “coronary artery bypass graft” OR “angina” OR “coronary artery disease” OR “coronary heart disease” OR “heart” OR “cardiac”)

#24 (#22 OR #23)

#25 (randomized AND controlled AND trial): pt

#27 (#3 AND #21 AND #24 AND #25)

**PsycINFO:**

S1 DE "Major Depression" OR TI depress*

S2 TX (“myocardial infarction” OR “acute coronary syndrome” OR “heart failure” OR “CABG” OR “coronary artery bypass graft” OR “angina” OR “coronary artery disease” OR “coronary heart disease” OR heart OR cardiac) or DE "Cardiovascular Diseases"

S3 MJ "Treatment" OR TI "Therapy" OR AB "Therapy" OR TI "Intervention" OR

AB "Intervention" OR TI Pharmacologic* OR AB Pharmacologic* OR TI

Antidepress* OR AB Antidepress* OR TI "Psychotherapy" OR AB "Psychotherapy" OR TI “collaborative care” OR AB “collaborative care” OR TI screen OR AB screen OR TI screening OR AB screening OR TI assessment OR AB assessment OR TI evaluation OR AB evaluation

S4 S1 AND S2 AND S3

*Limiters: Methodology – Treatment Outcome / Clinical Trial

**CINAHL:**

S1 ((MH "Depression") OR (MH “Depressive disorders”))

S2 ((MH "Cardiovascular diseases") OR (TX “myocardial infarction” OR “acute coronary syndrome” OR “heart failure” OR “CABG” OR “coronary artery bypass graft” OR “angina” OR “coronary artery disease” OR “coronary heart disease” OR “heart” OR “cardiac”))

S3 (MJ “drug therapy”) OR (MJ “psychotherapy”) OR TI (treatment OR therapy OR intervention OR “collaborative care” OR screen OR screening OR assessment OR evaluation) OR AB (treatment OR therapy OR intervention OR “collaborative care” OR screen OR screening OR assessment OR evaluation)

S4 S1 AND S2 AND S3 AND S4

* Limit to clinical trial, humans

**EMBASE:**

1. (depression/exp OR depressive)
2. (“Cardiovascular disease” OR “myocardial infarction” OR “acute coronary syndrome” OR “heart failure” OR CABG OR “coronary artery bypass graft” OR angina OR “coronary artery disease” OR “coronary heart disease” OR heart OR cardiac)
3. (therapy/mj OR “intervention study”/mj OR pharmacological:ti,ab OR “antidepressant agent”/mj OR antidepress*:ti,ab OR SSRI:ti,ab OR psychotherapy/mj OR treatment:ti,ab OR “collaborative care”:ti,ab OR screen:ti,ab OR screening:ti,ab OR assessment:ti,ab OR evaluation:ti,ab)
4. (random*:ti,ab)
5. 1 AND 2 AND 3 AND 4

* Map to preferred terminology, include sub-terms/derivatives (explosion search)

Limits: humans, controlled clinical trial, randomized controlled trial

**ISI**

1. TS=(Depression or Depressive disorders)
2. TS=(“Cardiovascular diseases” OR “cardiovascular disease” OR “myocardial infarction” OR “acute coronary syndrome” OR “heart failure” OR CABG OR “coronary artery bypass graft” OR angina OR “coronary artery disease” OR “coronary heart disease” OR heart OR cardiac)
3. TS=(Therapeutics OR therapy) OR TI=(drug therapy OR intervention OR treatment OR pharmacological OR psychological OR antidepress* OR psychotherapy OR cognitive OR behavior OR outcome OR effect* OR “collaborative care” OR screen OR screening OR assessment OR evaluation)
4. TS=(random*)
5. #1 AND #2 AND #3 AND #4

*Databases=SCI-EXPANDED, SSCI, A&HCI

**SCOPUS:**

(TITLE-ABS-KEY(depression OR depressive OR depressed)) AND (TITLE-ABS-KEY(“Cardiovascular disease” OR “myocardial infarction” OR “acute coronary syndrome” OR “heart failure” OR CABG OR “coronary artery bypass graft” OR angina OR “coronary artery disease” OR “coronary heart disease”)) AND ((TITLE(therapeutics)) OR (TITLE(therapy)) OR (TITLE-ABS-KEY(treatment)) OR (TITLE ("drug therapy")) OR (TITLE(intervention)) OR (TITLE(outcome*)) OR (TITLE(result*)) OR (TITLE(pharmacological)) OR (TITLE(trial)) OR (TITLE(antidepress*)) OR (TITLE(psychotherapy)) OR (TITLE (treatment)) OR (TITLE (psychologic*)) OR (TITLE(cognitive)) OR (TITLE(behavior)) OR (TITLE-ABS-KEY(“collaborative care”)) OR (TITLE-ABS-KEY(screen OR screening OR assessment OR evaluation))) AND (TITLE-ABS-KEY(random*)
