# Supplementary material for: Does Evidence Support the American Heart Association's Recommendation to Screen Patients for Depression in Cardiovascular Care? An Updated Systematic Review
Source: PLoS One. 2013 Jan 7;8(1):e52654. doi: 10.1371/journal.pone.0052654 (PMC3538724; doi:10.1371/journal.pone.0052654)
Supplement: File S2 — Relevant Systematic Reviews. (DOCX) [file pone.0052654.s002.docx]

**SUPPORTING INFORMATION 2. Relevant Systematic Reviews**

1. Barth J, Schumacher M, Herrmann-Lingen C. Depression as a risk factor for mortality in patients with coronary heart disease: a meta-analysis. Psychosom Med 2004;66:802-13.
2. Beltman MW, Voshaar RC, Speckens AE. Cognitive-behavioural therapy for depression in people with a somatic disease: meta-analysis of randomised controlled trials. Br J Psychiatry 2010;197:11-9.
3. Baumeister H, Hutter N, Bengel J. Psychological and pharmacological interventions for depression in patients with coronary artery disease. Cochrane Database Syst Rev 2011: CD008012.
4. Bohlmeijer E, Prenger R, Taal E, Cuijpers P. The effects of mindfulness-based stress reduction therapy on mental health of adults with a chronic medical disease: a meta-analysis. J Psychosom Res 2010;68:539-44.
5. Bush DE, Ziegelstein RC, Patel UV, et al. Post-Myocardial Infarction Depression. Summary, Evidence Report/Technology Assessment No. 123. (Prepared by the Johns Hopkins University Evidence-based Practice Center under Contract No. 290-02-0018.) AHRQ Publication No. 05-E018-1. Rockville, MD: Agency for Healthcare Research and Quality. May 2005.
6. Cimpean D, Drake RE. Treating co-morbid chronic medical conditions and anxiety/depression. Epidemiol Psychiatr Sci 2011;20;141-50.
7. Clark AM, Hartling L, Vandermeer B, McAlister FA. Meta-analysis: secondary prevention programs for patients with coronary artery disease. Ann Intern Med 2005;143:659-72.
8. Dekker RL. Cognitive behavioral therapy for depression in patients with heart failure: a critical review. Nurs Clin North Am 2008;43:155-70.
9. Dekker RL. Cognitive therapy for depression in patients with heart failure: a critical review. Heart Fail Clin 2011;7:127-41.
10. Delville CL, McDougall G. A systematic review of depression in adults with heart failure: instruments and incidence. Issues Ment Health Nurs 2008;29:1002-17.
11. Dowlati Y, Herrmann N, Swardfager WL, Reim EK, Lanctôt KL. Efficacy and tolerability of antidepressants for treatment of depression in coronary artery disease: a meta-analysis. Can J Psychiatry 2010;55:91-9.
12. Dusseldorp E, van Elderen T, Maes S, Meulman J, Kraaij V. A meta-analysis of psychoeducational programs for coronary heart disease patients. Health Psychol 1999;18:506-19.
13. Frasure-Smith N, Lespérance F. Reflections on depression as a cardiac risk factor. Psychosom Med 2005;67:S19-25.
14. Freedenberg V, Thomas SA, Friedmann E. Anxiety and depression in implanted cardioverter-defibrillator recipients and heart failure: a review. Heart Fail Clin 2011;7:59-68.
15. Goldston K, Baillie AJ. Depression and coronary heart disease: a review of the epidemiological evidence, explanatory mechanisms and management approaches. Clin Psychol Rev 2008;28:288-306.
16. Hasnain M, Vieweg WV, Lesnefsky EJ, Pandurangi AK. Depression screening in patients with coronary heart disease: a critical evaluation of the AHA guidelines. J Psychosom Res 2011;71:6-12.
17. Hemingway H, Marmot M. Evidence based cardiology: psychosocial factors in the aetiology and prognosis of coronary heart disease. Systematic review of prospective cohort studies. BMJ 1999;318:1460-7.
18. Kang-Yi CD, Gellis ZD. A systematic review of community-based health interventions on depression for older adults with heart disease. Aging Ment Health 2010;14:1-19.
19. Kuper H, Marmot H, Hemingway H. Systematic review of prospective cohort studies of psychosocial factors in the etiology and prognosis of coronary heart disease. Semin Vasc Med 2002;2:267-314.
20. Lane DA, Chong AY, Lip GYH. Psychological interventions for depression in heart failure. Cochrane Database Syst Rev 2005: CD003329.
21. Linden W, Phillips MJ, Leclerc J. Psychological treatment of cardiac patients: a meta-analysis. Eur Heart J 2007;28:2972-84.
22. Linden W, Stossel C, Maurice J. Psychosocial interventions for patients with coronary heart disease: a meta-analysis. Arch Intern Med 1996;156:745-52.
23. Magyar-Russell G, Thombs BD, Cai JX, et al. The prevalence of anxiety and depression in adults with implantable cardioverter defibrillators: a systematic review. J Psychosom Res 2011;71:223-31.
24. Mazza M, Lotrionte M, Biondi-Zoccai G, Abbate A, Sheiban I, Romagnoli E. Selective serotonin reuptake inhibitors provide significant lower re-hospitalization rates in patients recovering from acute coronary syndromes: evidence from a meta-analysis. J Psychopharmacol 2010;24:1785-92.
25. McCabe PJ. Psychological distress in patients diagnosed with atrial fibrillation: the state of the science. J Cardiovasc Nurs 2010;25:40-51.
26. McGillion M, Arthur H, Victor JC, Watt-Watson J, Cosman T. Effectiveness of psychoeducational interventions for improving symptoms, health-related quality of life, and psychological well being in patients with stable angina. Curr Cardiol Rev 2008;4:1-11.
27. Meijer A, Conradi HJ, Bos EH, Thombs BD, van Melle JP, de Jonge P. Prognostic association of depression following myocardial infarction with mortality and cardiovascular events: a meta-analysis of 25 years of research. Gen Hosp Psychiatry 2011;33:203-16.
28. Nicholson A, Kuper H, Hemingway H. Depression as an aetiologic and prognostic factor in coronary heart disease: a meta-analysis of 6362 events among 146 538 participants in 54 observational studies. Eur Heart J 2006;27:2763-74.
29. O’Neil A, Sanderson K, Oldenburg B, Taylor CB. Impact of depression treatment on mental and physical health-related quality of life of cardiac patients: a meta-analysis. J Cardiopulm Rehabil Prev.2011;31:146-56.
30. Pizzi C, Rutjes AW, Costa GM, Fontana F, Mezzetti A, Manzoli L. Meta-analysis of selective serotonin reuptake inhibitors in patients with depression and coronary heart disease. Am J Cardiol 2011;107:972-9.
31. Rayner L, Price A, Evans A, Valsraj K, Higginson IJ, Hotopf M. Antidepressants for depression in physically ill people. Cochrane Database Syst Rev.2010: CD007503.
32. Rees K, Bennett P, West R, Davey Smith G, Ebrahim S. Psychological interventions for coronary heart disease. Cochrane Database Syst Rev 2004:CD002902.
33. Regan KL. Depression treatment with selective serotonin reuptake inhibitors for the postacute coronary syndrome population: a literature review. J Cardiovasc Nurs 2008;23:489-96.
34. Rizzo M, Creed F, Goldberg D, Meader N, Pilling S. A systematic review of non-pharmacological treatments for depression in people with chronic physical health problems*.* J Psychosom Res 2011;71:18-27.
35. Rutledge T, Reis VA, Linke SE, Greenberg BH, Mills PJ. Depression in heart failure: a meta-analytic review of prevalence, intervention effects, and associations with clinical outcomes. J Am Coll Cardiol 2006;48:1527-37.
36. Shores MM, Pascualy M, Veith RC. Major depression and heart disease: treatment trials. Semin Clin Neuropsychiatry 1998;3:87-101.
37. Sorensen C, Friis-Hasche E, Haghfelt T, Bech P. Postmyocardial infarction mortality in relation to depression: a systematic critical review. Psychother Psychosom 2005;74:69-80.
38. Stafford L, Berk M, Reddy P, Jackson HJ. Comorbid depression and health-related quality of life in patients with coronary artery disease. J Psychosom Res 2007;62:401-10.
39. Stefanatou A, Kouris N, Lekakis J. Treatment of depression in elderly patients with cardiovascular disease: research data and future prospects. Hellenic J Cardiol 2010;51:142-52.
40. Summers KM, Martin KE, Watson K. Impact and clinical management of depression in patients with coronary artery disease. Pharmacotherapy 2010;30:304-22.
41. Taylor D, Meader N, Bird V, Pilling S, Creed F, Goldberg D. Pharmacological interventions for people with depression and chronic physical health problems: systematic review and meta-analyses of safety and efficacy. Br J Psychiatry 2011;198:179-88.
42. Thombs BD, Bass EB, Ford DE, et al. Prevalence of depression in survivors of acute myocardial infarction. J Gen Intern Med 2006;21:30-8.
43. Thombs BD, Magyar-Russell G, Bass EB, et al. Performance characteristics of depression screening instruments in survivors of acute myocardial infarction: review of the evidence. Psychosomatics 2007;48:185-94.
44. Tisminetzky M, Miozzo R, Goldberg R, McLaughlin T. Assessing depression in patients with an acute coronary syndrome: a literature review. Curr Psychiatry Rev 2011;7:217-25.
45. Van Dixhoorn J, White A. Relaxation therapy for rehabilitation and prevention in ischaemic heart disease: a systematic review and meta-analysis. Eur J Cardiovasc Prev Rehabil 2005;2:193-202.
46. van Melle JP, de Jonge P, Spijkerman TA, et al. Prognostic association of depression following myocardial infarction with mortality and cardiovascular events: a meta-analysis. Psychosom Med 2004;66:814-22.
47. Von Ruden AE, Adson DE, Kotlyar M. Effect of selective serotonin reuptake inhibitors on cardiovascular morbidity and mortality. J Cardiovasc Pharmacol Ther 2008;13:32-40.
48. Watson K, Summers KM. Depression in patients with heart failure: clinical implications and management. Pharmacotherapy 2009;29:49-63.
49. Welton NJ, Caldwell DM, Adamopoulos E, Vedhara K. Mixed treatment comparison meta-analysis of complex interventions: psychological interventions in coronary heart disease. Am J Epidemiol 2009;169:1158-65.
50. Whalley B, Rees K, Davies P, et al. Psychological interventions for coronary heart disease. Cochrane Database Syst Rev 2011: CD002902.
51. Zuidersma M, Thombs BD, de Jonge P. Onset and recurrence of depression as predictors of cardiovascular prognosis in depressed acute coronary syndrome patients: a systematic review. Psychother Psychosom 2011; 80:227-37.
