# Supplementary material for: Does Evidence Support the American Heart Association's Recommendation to Screen Patients for Depression in Cardiovascular Care? An Updated Systematic Review
Source: PLoS One. 2013 Jan 7;8(1):e52654. doi: 10.1371/journal.pone.0052654 (PMC3538724; doi:10.1371/journal.pone.0052654)
Supplement: File S3 — Journals Included in Manual Searching. (DOCX) [file pone.0052654.s003.docx]

**SUPPORTING INFORMATION 3. Journals Included in Manual Searching**

American Heart Journal

American Journal of Cardiology

American Journal of Medicine

American Journal of Geriatric Psychiatry

American Journal of Psychiatry

Annals of Behavioral Medicine

Annals of Internal Medicine

Archives of General Psychiatry

Archives of Internal Medicine

Biological Psychiatry

British Medical Journal

Canadian Journal of Psychiatry

Canadian Medical Association Journal

Circulation

European Heart Journal

European Journal of Heart Failure

European Journal of Preventive Cardiology

General Hospital Psychiatry

Health Psychology

Heart

Herz

JAMA

Journal of Affective Disorders

Journal of Behavioral Medicine

Journal of Cardiopulmonary Rehabilitation and Prevention

Journal of the American College of Cardiology

Journal of General Internal Medicine

Journal of Psychosomatic Research

Lancet

New England Journal of Medicine

Psychosomatic Medicine

Psychosomatics

Psychotherapy and Psychosomatics
