# Supplementary material for: Does Evidence Support the American Heart Association's Recommendation to Screen Patients for Depression in Cardiovascular Care? An Updated Systematic Review
Source: PLoS One. 2013 Jan 7;8(1):e52654. doi: 10.1371/journal.pone.0052654 (PMC3538724; doi:10.1371/journal.pone.0052654)
Supplement: File S4 — Variables Included in Data Extraction Forms. (DOCX) [file pone.0052654.s004.docx]

**SUPPORTING INFORMATION 4. Variables Included in Data Extraction Forms**

**Variables Included in Data Extraction Form for Key Question #1**

First author

Year

Country

Diagnosis/procedure

Number of patients

Inpatient/outpatient assessment

Key inclusion criteria

Key exclusion criteria

Recruitment rate of eligible patients

Mean age

Percent male

Structured interview used

Timing of assessment with structured interview relative to acute event (if applicable)

Number (%) with major depressive disorder

Screening tool and cutoff threshold

Derivation of cutoff (e.g., literature, exploratory)

Range of cutoffs reported

Timing of assessment with screening tool relative to acute event (if applicable)

Number (%) above threshold on screening tool

Interviewer access to screening results?

Order of administration (structured interview versus screening tool)

Number positive MDD/positive screening tool

Number positive MDD/negative screening tool

Number negative MDD/positive screening tool

Number negative MDD/negative screening tool

Sensitivity

Specificity

Positive predictive value

Negative predictive value

Notes

**Variables Included in Data Extraction Form for Key Question #2**

First author

Year

Country

Published trial design preceded results?

Trial registration (registration requirement, registration status, registration number)

Study funding source

Number of authors with disclosed conflicts of interest / total number of authors

Diagnosis/procedure

Key inclusion criteria

Key exclusion criteria

Mean age

Percent male

Structured interview used

Timing of assessment and enrolment in trial relative to acute event (if applicable)

Treatment

Treatment protocol (e.g., dose, hours per week)

Control group (e.g., UC, placebo, supportive therapy)

Weeks of treatment

N intent to treat

N treatment

N control

For each depression continuous outcome variable:

Outcome variable

Effect size measure (e.g., d, g, r)

Effect size (+ for treatment better)

Intent to treat?

Follow-up duration for cardiac outcomes

For each cardiac outcome (e.g., mortality, composite events)

Outcome definition

Treatment group – N evaluated

Treatment group – N with outcome

Control group – N evaluated

Control group – N with outcome

Intent to treat?

**No Data Extracted for Key Question #3 – No Eligible Studies**
