# Supplementary material for: Does Evidence Support the American Heart Association's Recommendation to Screen Patients for Depression in Cardiovascular Care? An Updated Systematic Review
Source: PLoS One. 2013 Jan 7;8(1):e52654. doi: 10.1371/journal.pone.0052654 (PMC3538724; doi:10.1371/journal.pone.0052654)
Supplement: File S5 — QUADAS-2 Risk of Bias and Applicability Judgments. (DOCX) [file pone.0052654.s005.docx]

**SUPPORTING INFORMATION 5. QUADAS-2 Risk of Bias and Applicability Judgments**

| **Domain** | **Signaling Questions*** | **Risk of Bias†** | **Applicability Concerns†** |
| --- | --- | --- | --- |
| Patient Selection | (1) Was a consecutive or random sample of patients enrolled?‡  (2) Was a case-control design avoided?  (3) Did the study avoid inappropriate exclusions? | Could the selection of patients have introduced bias? | Are there concerns that the included patients and setting do not match the review question?§ |
| Index Test | (1) Were the index test results interpreted without knowledge of the results of the reference standard?\|\|  (2) If a threshold was used, was it pre-specified?¶ | Could the conduct or interpretation of the index test have introduced bias? | Are there concerns that the index test, its conduct, or interpretation differ from the review question? |
| Reference Standard | (1) Is the reference standard likely to correctly classify the target condition?#  (2) Were the reference standard results interpreted without knowledge of the results of the index test? | Could the reference standard, its conduct, or its interpretation have introduced bias? | Are there concerns that the target condition as defined by the reference standard does not match the question?** |
| Flow and Timing | (1) Was there an appropriate interval between index test and reference standard?††  (2) Did all patients receive a reference standard?‡‡  (3) Did all patients receive the same reference standard?  (4) Were all patients included in the analysis? | Could the patient flow have introduced bias? | Not applicable |

* Signaling questions are rated “yes”, “no”, or “unclear”. **†** Risk of bias judgments and concerns related to applicability are rated “high”, “low” or “unclear”. ‡ Rated “yes” if a consecutive or random sample of patients were recruited for the study, “no” if a very low rate of eligible patients were included in the study, and “unclear” if the study indicates that consecutive patients were included, but does not specify the total number of eligible patients and the number consented. § Rated “high” if study includes patients with already diagnosed or treated depression, as they would not be screened or constitute newly identified cases in clinical practice, and “unclear” if the study does not exclude patients with already diagnosed or treated depression, but does not specify the number of such patients that were included. Studies were not downgraded for sampling only one type of CHD diagnosis or procedure. || This signalling question was omitted from the quality assessment, as the interpretation of the index test is fully automated in scoring self-report depressive symptom questionnaires. ¶ Rated “yes” if the study only tested a standard cutoff of the screening questionnaire, and the objective of the study was not the performance of the screening questionnaire at different cutoffs. # Rated “yes” for all studies as the systematic review inclusion criteria required the reference standard to be a standardized structured or semi-structured diagnostic interview based on DSM or ICD criteria. ** Rated “unclear” or “high” if the study indicated that the standardized structured or semi-structured diagnostic interview and/or DSM or ICD diagnostic criteria were modified, depending on the extent and nature of the modifications. †† Rated “yes” if the index test and reference standard were administered within 1 week of each other, “no” if longer, and “unclear” if not specified in the published report. Studies in which some patients were administered the index text and reference standard more than 2 weeks apart were excluded. When the interval between the administration of the screening instrument and the diagnostic interview was not specified in the published report, study authors were contacted for clarification to determine study eligibility. Quality assessment ratings, however, were based only on published information. ‡‡ This signaling question was added, and rated “no” if all positive screens, but only a random sample of negative screens, were administered the reference standard, since the index score influenced the likelihood of receiving the reference standard.

**Reference:** Whiting PF, Rutjes AW, Westwood ME, et al. QUADAS-2: a revised tool for the quality assessment of diagnostic accuracy studies. *Ann Intern Med.* 2011;155:529-536.
