# Supplementary material for: Does Evidence Support the American Heart Association's Recommendation to Screen Patients for Depression in Cardiovascular Care? An Updated Systematic Review
Source: PLoS One. 2013 Jan 7;8(1):e52654. doi: 10.1371/journal.pone.0052654 (PMC3538724; doi:10.1371/journal.pone.0052654)
Supplement: File S6 — Cochrane Risk of Bias Tool Domains. (DOCX) [file pone.0052654.s006.docx]

**SUPPORTING INFORMATION 6. Cochrane Risk of Bias Tool Domains**

**Sequence generation:** Describe the method used to generate the allocation sequence in sufficient detail to allow an assessment of whether it should produce comparable groups.

**Allocation concealment** Describe the method used to conceal the allocation sequence in sufficient detail to determine whether intervention allocations could have been foreseen in advance of, or during, enrolment.

**Blinding of participants, personnel and outcome assessors** *Assessments should be made for each main outcome (or class of outcomes).* Describe all measures used, if any, to blind study participants and personnel from knowledge of which intervention a participant received. Provide any information relating to whether the intended blinding was effective.

**Incomplete outcome data** *Assessments should be made for each main outcome (or class of outcomes).* Describe the completeness of outcome data for each main outcome, including attrition and exclusions from the analysis. State whether attrition and exclusions were reported, the numbers in each intervention group (compared with total randomized participants), reasons for attrition/exclusions where reported, and any re-inclusions in analyses performed by the review authors.

**Selective outcome reporting** State how the possibility of selective outcome reporting was examined by the review authors, and what was found.

**Pharmaceutical industry funding** State the funding source(s) of the trial, or indicate if the trial funding source was not reported.

**Author-industry financial ties and/or employment** State whether any trial authors disclosed financial ties and/or employment by the pharmaceutical industry, or if author-industry financial ties or affiliation were not reported.

**Other sources of bias** State any important concerns about bias not addressed in the other domains in the tool. If particular questions/entries were pre-specified in the review’s protocol, responses should be provided for each question/entry.

**Reference:** Higgins JPT, Altman DG, Sterne JAC, editors. Chapter 8: Assessing risk of bias in included studies. In: Higgins JPT, Green S, editors. *Cochrane Handbook for Systematic Reviews of Interventions* Version 5.1.0 [updated March 2011]. The Cochrane Collaboration, 2011. <http://www.cochrane-handbook.org>. Accessed June 1, 2012.
