# Supplementary material for: Does Evidence Support the American Heart Association's Recommendation to Screen Patients for Depression in Cardiovascular Care? An Updated Systematic Review
Source: PLoS One. 2013 Jan 7;8(1):e52654. doi: 10.1371/journal.pone.0052654 (PMC3538724; doi:10.1371/journal.pone.0052654)
Supplement: File S7 — Quality Assessment of Studies of Diagnostic Accuracy (QUADAS-2). (DOCX) [file pone.0052654.s007.docx]

**SUPPORTING INFORMATION 7. Quality Assessment of Studies** **of Diagnostic Accuracy (QUADAS-2)**

|  | **QUADAS-2 Domains*** | | | | | | | |
| --- | --- | --- | --- | --- | --- | --- | --- | --- |
|  | **Risk of Bias** | | | |  | **Applicability Concerns** | | |
| **First Author,**  **Year,**  **Country** | **Patient Selection** | **Index Test** | **Reference Standard** | **Flow and Timing** |  | **Patient Selection** | **Index Test** | **Reference Standard** |
| Bunevicius,  2012,  Lithuania [17] | Low | High | Low | Low |  | Low | Low | Low |
| Cruz,  2010,  Brazil [8] | Unclear | Low | Low | Low |  | High | Low | Low |
| Dickens,  2004,  United Kingdom [18] | Low | High | Unclear | Unclear |  | High | Low | Low |
| Frasure-Smith,  1995,  Canada [19] | Low | Low | Unclear | Low |  | Unclear | Low | Unclear |
| Frasure-Smith,  2008,  Canada [9] | High | Low | Low | Low |  | High | Low | Low |
| Freedland,  2003,  United States [20] | High | Low | Unclear | Low |  | Unclear | Low | Low |
| Gutierrez,  1999,  Canada [21] | High | Unclear | Low | Low |  | Unclear | Low | Low |
| **Heart and Soul** |  |  |  |  |  |  |  |  |
| McManus,  2005,  United States [22] | High | Low | Low† | Low |  | High | Low | Low |
| Thombs,  2008,  United States [11] | High | High | Low | Low |  | High‡ | Low | Low |
| Elderon,  2011,  United States [10] | High§ | Low | Low† | Low |  | High | Low | Low |
| **Huffman** |  |  |  |  |  |  |  |  |
| Huffman,  2006,  United States [23] | Low | High | High | Low |  | High | Low | Low |
| Huffman,  2010,  United States [12] | Low | High | High | Low |  | High | Low | Low |
| Jacq,  2009,  France [13] | Low | Low | Unclear | Unclear |  | High | Low | Low |
|  |  |  |  |  |  |  |  |  |
| Low,  2007,  Canada [24] | Unclear | High | Low | Low |  | High | Low | Low |
| Pinho,  2010,  Brazil [14] | Unclear | High | Low | High |  | High | Low | Low |
| Stafford,  2007,  Australia [25] | High | High | Low | Low |  | Unclear | Low | Low |
| Swardfager,  2011,  Canada [16] | High | Low | Unclear | Unclear |  | High | Low | Low |
| Tiringer,  2008,  Hungary [15] | Unclear | High | Low | Unclear |  | High | Low | Low |

* See Appendix 5 for QUADAS-2 risk of bias and applicability judgments. Items are rated “low”, “high” and “unclear” based on the QUADAS-2 guidelines and reflect the risk of bias or the degree of concern about applicability. Quality ratings were based only on published information. QUADAS-2 coding notes are available from the corresponding author. † The blinding of interviewers to the results of the screening instrument was reported for this cohort in Thombs, 2008 [11]. ‡ The number of included patients already treated for depression in this cohort was reported in McManus, 2005 [22] and Elderon, 2011 [10]. § Information on the rate of recruitment for this cohort was reported in McManus, 2005 [22] and Thombs, 2008 [11].
