# Supplementary material for: Does Evidence Support the American Heart Association's Recommendation to Screen Patients for Depression in Cardiovascular Care? An Updated Systematic Review
Source: PLoS One. 2013 Jan 7;8(1):e52654. doi: 10.1371/journal.pone.0052654 (PMC3538724; doi:10.1371/journal.pone.0052654)
Supplement: File S8 — Assessment of Risk of Bias in Randomized Controlled Trials in Key Question #2 (Treatment). (DOCX) [file pone.0052654.s008.docx]

**SUPPORTING INFORMATION 8. Assessment of Risk of Bias in Randomized Controlled Trials in Key Question #2 (Treatment)**

|  | **Cochrane Risk of Bias Tool Domains*** | | | | | | | | |
| --- | --- | --- | --- | --- | --- | --- | --- | --- | --- |
| **Trial,**  **Year,**  **Country** | **Random**  **Sequence Generation** | **Allocation Concealment** | **Blinding of Participants and Personnel** | **Blinding of Outcome Assessment†** | **Incomplete Outcome Data†** | **Selective Outcome Reporting** | **Pharmaceutical Industry Funding‡** | **Author-Industry Financial Ties and/or Industry Employment‡** | **Other Sources of Bias** |
| CREATE, 2007, Canada – Citalopram vs. placebo [30] | Low | Low | Low | Observer-rated: Low  Self-report: Low  CHD: Low | Observer-rated: Low  Self-report: Low  CHD: Low | Low | Low | Unclear | Low |
| CREATE, 2007, Canada – IPT + CM vs. CM [30] | Low | Low | High | Observer-rated: Low  Self-report: High  CHD: Low | Observer-rated: Low  Self-report: Low  CHD Low | Low | Low | Low | Low |
| ENRICHD, 2003, United States [31] | Low | Low | High | Observer-rated: Unclear  Self-report: High  CHD: Low | Observer-rated: Low  Self-report: Low  CHD: Low | Low | Low | Low | Low |
| Fraguas, 2009,  Brazil [28] | Unclear | Unclear | Low | Observer-rated: Unclear  Self-report: NA  CHD: NA | Observer-rated: Low  Self-report: NA  CHD: NA | Unclear | Low | Unclear | Unclear§ |
| Honig, 2007,\|\| Netherlands [32] | Low | Low | Low | Observer-rated: Unclear  Self-report: Low  CHD: Low | Observer-rated: Low  Self-report: Low  CHD: Low | Unclear | High | Unclear¶ | Low |
| MIND-IT, 2007,\|\| Netherlands [33] | Low | Low | High | Observer-rated: NA  Self-report: High  CHD: Low | Observer-rated: NA  Self-report: High  CHD: Low | Low | Low | Low | High# |
| SADHART, 2002, United States, Canada, Europe, Australia [34] | Unclear | Unclear | Low | Observer-rated: Unclear  Self-report: NA  CHD: Low | Observer-rated: Low  Self-report: Low  CHD: Low | Unclear | High | High | Low |
| SADHART-CHF, 2010, United States [29] | Low | Low | Low | Observer-rated: Unclear  Self-report: NA  CHD: Low | Observer-rated: Low  Self-report: NA  CHD: Low | Low | Low | Low | Low |
| Strik, 2000, Netherlands [35] | Unclear | Unclear | Low | Observer-rated: Unclear  Self-report: NA  CHD: Unclear | Observer-rated: Low  Self-report: NA  CHD: Low | Unclear | High | High | Low |

* See Appendix 6 for domain descriptions. Domains are scored as ‘high’, ‘low’, or ‘uncertain’ risk of bias. Risk of bias ratings were based only on published information. Coding notes are available from the corresponding author. † Ratings are provided for observer-rated depression scales, self-report depression questionnaires, and cardiovascular outcomes. ‡ Additional domain added to standard Cochrane Risk of Bias tool. § Trial halted after an unplanned interim analysis of a small number of patients (total N = 37) showed a high placebo response. It is possible that early stopping could have actually reduced the effect that would have been found, although it is unknown whether or not this is case. || The Honig, 2007 [32] study was an RCT nested within the MIND-IT study [33]. ¶ Author-industry financial ties were not reported. The authors of the MIND-IT trial [33], of which the Honig, 2007 study [32] was a nested RCT, declared no conflicts of interest. However, of the 12 authors of the Honig, 2007 study [32] only 9 were authors of the published MIND-IT report [33]. # Reported depression outcomes were assessed at 18 months post-myocardial infarction (0-9 months after completion of treatment), and breakdown of timing of assessment was not reported.

CHD = coronary heart disease; CREATE = Canadian Cardiac Randomized Evaluation of Antidepressant and Psychotherapy Efficacy trial; ENRICHD = Enhancing Recovery in Coronary Heart Disease Patients; MIND-IT = Myocardial Infarction and Depression-Intervention trial; NA = not applicable; SADHART = Sertraline Antidepressant Heart Attack Randomized trial; SADHART-CHF = Sertraline Against Depression and Heart Disease in Chronic Heart Failure
