# Supplementary material for: Does Evidence Support the American Heart Association's Recommendation to Screen Patients for Depression in Cardiovascular Care? An Updated Systematic Review
Source: PLoS One. 2013 Jan 7;8(1):e52654. doi: 10.1371/journal.pone.0052654 (PMC3538724; doi:10.1371/journal.pone.0052654)
Supplement: File S9 — Excluded Studies for Effect of Screening on Depression Outcomes (Key Question #3). (DOCX) [file pone.0052654.s009.docx]

**SUPPORTING INFORMATION 9. Excluded Studies for Effect of Screening on Depression Outcomes (Key Question #3)**

| **First Author,**  **Year,**  **Country** | **Diagnosis/**  **Setting** | **N Consented/ Randomized*** | **Comparison** | **Depression Outcomes** | **Reason(s) for exclusion** |
| --- | --- | --- | --- | --- | --- |
| Annunziato,  2008,  United States^1^ | Outpatient cardiology clinic | 316 | **Intervention**: Patients presenting to the cardiology clinic completed the PHQ-9 and IES. A positive screening result on the PHQ-9 (≥ 15) or the IES prompted an evaluation by the cardiologist or nurse-practitioner. In Phase I, patients with positive screens were referred to outpatient psychiatry. In Phase II, patients with a positive screen were seen by a psychiatrist placed in the cardiology clinic.  **Control**: NA (no comparison group). | Only number of positive screens, number referred for psychiatric evaluation, and number referred to psychiatry and seen by psychiatrist, but no depression outcomes, were assessed. Of 316 patients screened, only 20 (0.6%) screened positive (≥ 15) on the PHQ-9. None of 12 patients with positive screens who were referred to outpatient psychiatry followed-up. | Not a RCT. Outcomes included number of positive screens and number referred and seen for psychiatric evaluation, but no depression outcomes were assessed. |
| Davidson,  2010,  United States^2^ | ACS | 237 | Patients with a BDI score ≥ 10 within 1 week of hospitalization for ACS and 3 months later were eligible for inclusion in the RCT of collaborative depression care.  **Intervention**: Collaborative care delivered by a clinical nurse specialist, psychologist, social worker, and/or psychiatrist. Initial patient choice of problem-solving therapy and/or pharmacotherapy, followed by a stepped-care approach, dependent on symptom severity.  **Control**: Usual care, with physicians informed of patients’ depression status. | Significantly greater decrease in BDI scores in the intervention group than control group. | Not a RCT of screening for depression. Patients were required to have persistent depressive symptoms to be eligible for the trial. In addition, only patients with depressive symptoms in the intervention group received a collaborative care intervention for depression, whereas patients with depressive symptoms in the control group received usual care. Thus, the trial did not evaluate whether patients who receive screening will have better depression outcomes than patients who are not screened when the same treatment resources are made available to both groups. |
| Frasure-Smith,  1997,  Canada^3^ | Post-AMI | 1376 | **Intervention**: Telephone screening beginning with the 20-item GHQ beginning 1 week after discharge from hospital and repeated at 1-month intervals for 1 year. Project nurses made follow-up telephone calls to all patients with GHQ ≥ 5 to assess psychological distress and provided home psychosocial interventions.  **Control**: Usual care. | No significant difference between groups for change in BDI scores at 1-year. | Screening was for distress, broadly, not MDD. The follow-up intervention for positive screens was geared towards distress, not MDD specifically. |
| Huffman,  2011,  United States^4^ | AMI, HF, UA, or arrhythmia | 175 | Patients admitted for acute cardiac disease were screened with the PHQ-2, and positive-screen patients (PHQ-2 ≥ 3) were screened with the PHQ-9. Patients with PHQ-9 scores ≥ 10, with 5 or more symptoms, including either depressed mood or anhedonia, were eligible for inclusion in the collaborative depression care RCT.  **Intervention**: Collaborative care intervention for depression, including patient education and treatment coordination, delivered by social work care managers, in consultation with the study psychiatrist and medical care providers, in hospital and over a 12-week post-discharge period.  **Control**: Usual care, with feedback on depression status to care providers. | Significantly greater decrease in PHQ-9 scores at 6 weeks and 12 weeks in the intervention group than control group. | Not a RCT of screening for depression. Patients were required to have depressive symptoms to be eligible for the trial. In addition, only patients with depressive symptoms in the intervention group received a collaborative care intervention for depression, whereas patients with depressive symptoms in the control group received usual care. Thus, the trial did not evaluate whether patients who receive screening will have better depression outcomes than patients who are not screened, when the same treatment resources are made available to both groups. |
| Norris,  2009,  Canada^5^ | CHD, post-cardiac catherization | 98† | Patients with a baseline CES-D ≥ 10 were eligible for inclusion in the RCT.  **Information and Telephone Follow-Up Intervention**: Patients received information by mail regarding community- and hospital-based mental health resources, and were also contacted by phone by a nurse to discuss the written information.  **Information Intervention**: Patients received information by mail regarding community- and hospital-based mental health resources.  **Control:** Usual care. | Significantly greater decrease in CES-D scores at 6 weeks and 12 weeks in both intervention groups compared to the control group. No difference between intervention groups. | Not a RCT of screening for depression. Patients were required to have depressive symptoms to be eligible for the trial. In addition, only patients with depressive symptoms in the intervention groups received information on mental health resources and telephone follow-up, whereas patients with depressive symptoms in the control group received usual care. Thus, the trial did not evaluate whether patients who receive screening will have better depression outcomes than patients who are not screened, when the same treatment resources are made available to both groups. |
| Rollman,  2009,  United States^6^ | Post-CABG | 302 | Patients were screened with the yes/no PHQ-2 prior to hospital discharge, and positive-screen patients (yes/no PHQ-2 ≥ 1) were administered the PHQ-9 at 2 weeks post-discharge from hospital. Patients with PHQ-9 scores ≥ 10 were eligible for inclusion in the RCT of collaborative depression care.  **Intervention**: Telephone-delivered collaborative care for depression, including patient education and treatment coordination, provided by nurse care managers, in consultation with primary care physicians and study psychiatrist, for 8 months.  **Control**: Usual care, with patients and primary care physicians informed of patients’ depression status. | Significantly greater decrease in SF-36 MCS and HAMD-17 scores at 8 months in the intervention group than control group. | Not a RCT of screening for depression. Patients were required to have depressive symptoms to be eligible for the trial. In addition, only patients with depressive symptoms in the intervention group received a collaborative care intervention for depression, whereas patients with depressive symptoms in the control group received usual care. Thus, the trial did not evaluate whether patients who receive screening will have better depression outcomes than patients who are not screened, when the same treatment resources are made available to both groups. |
| Smolderen,  2011,  United States^7^ | Post-AMI | 4,036‡ | **Intervention:** A 2-step depression screening protocol, similar to the AHA-recommended screening method, was implemented as part of usual clinical care for hospitalized AMI patients at a single center. Patients were screened by nursing staff with the PHQ-2, and those with positive screens (PHQ-2 ≥ 1) were screened with the PHQ-9. For patients with a PHQ-9 ≥ 5, inpatient physicians were notified, and outpatient physicians were notified about the need to follow-up. In addition, for patients with a PHQ-9 ≥ 10, depression treatment order forms were placed in patient’s charts.  **Control**: In parallel with the intervention described above, a prospective registry of AMI patient outcomes was conducted at the intervention hospital and 23 other centers without a screening protocol. Patients were screened with the PHQ-9 by trained data collectors. | Only rates of depression screening, number of positive screens, and rates of depression recognition by physicians based on patient charts, but no depression outcomes, were assessed. Of the 368 patients screened per protocol at the intervention center, only 22 (6%) had a PHQ-9 score ≥ 10. It is unlikely that all of these constituted newly-identified cases, as 40 of the 368 patients (11%) screened per the intervention protocol were already receiving treatment for depression. There was no significant difference in the rates of depression recognition based on chart notes between the intervention center and the 24 registry centers (including the intervention center). | Not a RCT. In addition, outcomes included rates of depression screening, number of positive screens, and rates of depression recognition by physicians, but no depression outcomes were assessed. |
| Sowden,§  2010,  United States^8^ | Inpatient cardiac units | 3,504\|\| | **Intervention:** Systematic depression screening using the AHA-recommended 2-step screening method was implemented as part of usual clinical care on 3 cardiac inpatient units. Patients were screened with the PHQ-2 at admission, and those with positive screens (PHQ-2 ≥ 3) and a diagnosis of AMI, HF, UA, or arrhythmia^c^ were verbally administered the PHQ-9.  **Control:** NA (no comparison group) | Only rates of depression screening, number of positive and negative screens for both instruments, and staff satisfaction with screening process, but no depression outcomes, were assessed. Of 3,504 patients screened with the PHQ-2, only 140 (0.4%) scored ≥ 10 on the PHQ-9. It is not known whether all of these constituted newly-identified cases, as patients with already diagnosed or treated depression were not excluded. | Not a RCT (single prospective cohort design). A positive depression screen based on a defined cutoff score on the PHQ-2 was used to determine who received further assessment with the PHQ-9, but a positive screen on the PHQ-9 was not used to determine further assessment or treatment. In addition, no depression outcomes were assessed. |
| Subramanian,  2008  United Kingdom,^9^ | IHD or diabetes | 365¶ | **Intervention:** Screening for depression in patients with IHD and diabetes with the “two-plus-one” questionnaire# was added to the United Kingdom Quality and Outcomes Framework, which provides financial incentives to general practitioners to improve chronic disease management. The present study consisted of a chart review in a single semi-rural general practice approximately a year after the introduction of depression screening to this framework.  **Control:** NA (no comparison group) | Only rates of depression screening, number of positive screens, and rates of depression diagnosis were assessed. Of the 365 patients screened, 27 were already diagnosed or receiving treatment for depression. Excluding these patients, only 3 patients were positive screens. Of these, one was lost to follow-up, and the other 2 were not diagnosed with depression. Thus, no new cases of depression were identified through screening, and it was not possible to assess depression outcomes. | Not a RCT. Data for IHD patients were not reported separately. In addition, only rates of depression screening, number of positive screens, and rates of depression diagnosis were assessed. It was not possible to assess depression outcomes, as no new cases of depression were identified through screening. |

ACS = acute coronary syndrome; AHA = American Heart Association; AMI = acute myocardial infarction; CABG = coronary artery bypass graft surgery; CES-D = Center for Epidemiological Studies Depression Scale; CHD = coronary heart disease; IHD = ischemic heart disease; BDI = Beck Depression Inventory; HF = heart failure; IES = Impact of Events Scale; NA = not applicable; PHQ-2 = Patient Health Questionnaire – 2; PHQ-9 = Patient Health Questionnaire – 9; RCT = randomized controlled trial; UA = unstable angina.

* Number consented for non-randomized controlled trials and number randomized for randomized controlled trials. †Number of patients who were randomized, and had complete baseline and 6-week CES-D scores. ‡ 503 patients at the intervention center were eligible for the intervention screening protocol, and had concurrent registry data. PHQ-9 data were available from 3,533 registry patients from the other 23 comparison centers. The number of patients consented was not reported. § Study methods and cohort linked to randomized trial of collaborative care for depression.^77^ || Number screened for depression with PHQ-2 out of 4,783 eligible patients. The number consented was not reported. ¶ 365 of 435 eligible patients were screened. The number consented was not reported. # 3-item yes/no questionnaire, consisting of two depressive symptom screening questions and, if either of the screening questions is answered affirmatively, followed by a question on interest in receiving help.

**REFERENCES – APPENDIX 9**

1. Annunziato RA, Rubinstein D, Sheikh S, et al. Site matters: winning the hearts and minds of patients in a cardiology clinic. Psychosomatics 2008;49:386-91.

2. Davidson KW, Rieckmann N, Clemow L, et al. Enhanced depression care for patients with acute coronary syndrome and persistent depressive symptoms: Coronary Psychosocial Evaluation Studies Randomized Controlled Trial. Arch Intern Med 2010;170:600-8.

3. Frasure-Smith N, Lesperance F, Prince RH, et al. Randomised trial of home-based psychosocial nursing intervention for patients recovering from myocardial infarction. Lancet 1997;350:473-9.

4. Huffman JC, Mastromauro CA, Gillian S, Fricchione GL, Healy BC, Januzzi JL. Impact of a depression care management program for hospitalized cardiac patients. Circ Cardiovasc Qual Outcomes 2011;4:198-205.

5. Norris CM, Patterson L, Galbraith D, Hegadoren KM. All you have to do is call: a pilot study to improve the outcomes of patients with coronary artery disease. Appl Nurs Res 2009;22:133-7.

6. Rollman BL, Belnap BH, LeMenager MS, et al. Telephone-delivered collaborative care for treating post-CABG depression: a randomized controlled trial. JAMA 2009;302:2095-103.

7. Smolderen KG, Buchanan DM, Amin AA, et al. Real-world lessons from the implementation of a depression screening protocol in acute myocardial infarction patients: implications for the American Heart Association depression screening advisory. Circ Cardiovasc Qual Outcomes 2011;4:283-92.

8. Sowden G, Mastromauro CA, Januzzi JL, Fricchione GL, Huffman JC. Detection of depression in cardiac inpatients: feasibility and results of systematic screening. Am Heart J 2010;159:780-7.

9. Subramanian DN, Hopayian K. An audit of the first year of screening for depression in patients with diabetes and ischaemic heart disease under the Quality and Outcomes Framework. Qual Prim Care 2008;16:341-4.
